# Optimizing Point-in-Space Continuous Monitoring System Sensor Placement on Oil and Gas Sites

**DOI:** 10.1021/acssusresmgt.4c00333

**Published:** 2024-12-18

**Authors:** Meng Jia, Troy Robert Sorensen, Dorit Martina Hammerling

**Affiliations:** † Department of Applied Mathematics and Statistics, Colorado School of Mines, Golden, Colorado 80401, United States; ‡ Energy Emissions Modeling and Data Lab, The University of Texas at Austin, Austin, Texas 78712, United States

**Keywords:** methane emission reduction, oil and gas, continuous monitoring systems, sensor placement, genetic algorithms, Pareto optimization

## Abstract

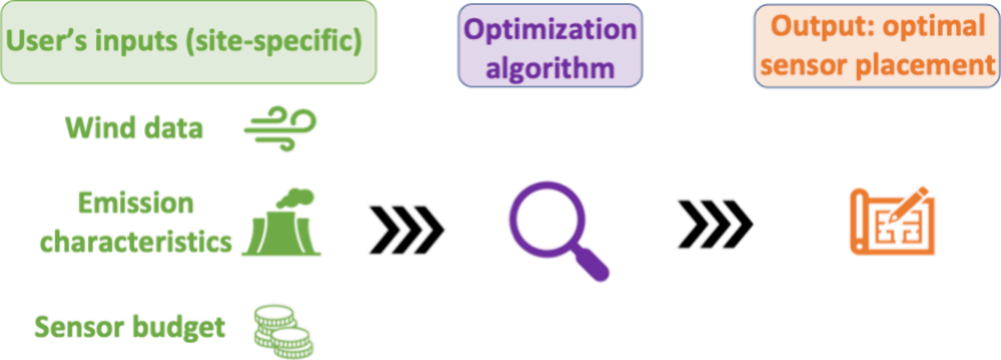

We propose a generic, modular framework to optimize the placement of point-in-space continuous monitoring system sensors on oil and gas sites aiming to maximize the methane emission detection efficiency. Our proposed framework substantially expands the problem scale compared to previous related studies and can be adapted for different objectives in sensor placement. This optimization framework is comprised of five steps: (1) simulate emission scenarios using site-specific wind and emission information; (2) set possible sensor locations under consideration of the site layout and any site-specific constraints; (3) simulate methane concentrations for each pair of emission scenario and possible sensor location; (4) determine emissions detection based on the site-specific simulated concentrations; and (5) select the best subset of sensor locations, under a given number of sensors to place, using genetic algorithms combined with Pareto optimization. We demonstrate the practicality and effectiveness of our framework through its application to an oil and gas emission testing facility with a large search space of possible sensor locations; a setting which is computationally infeasible to solve with commonly used mixed-integer linear programming. Additionally, a case study illustrates the successful application of our algorithm to an operating oil and gas site, showcasing its real-world applicability and effectiveness.

## Abstract 2

We developed a sensor placement optimization framework for continuous monitoring systems for point-in-space methane emissions detection on oil and gas sites.

## Introduction

Mitigating anthropogenic methane emissions is pivotal for achieving the goal of limiting increase in the global average temperature to below 1.5°C above pre-industrial levels set by the 2015 Paris Climate Agreement.^1,2^ Oil and gas emerges as a key sector for reducing emissions, contributing 22% of global anthropogenic methane emissions^3,4^ and 32% in the United States.^5^ In June 2024, the U.S. Environmental Protection Agency (EPA) and the U.S. Department of Energy (DOE) announced $850 million in federal funding aimed at reducing methane emissions from the oil and gas sector.^6^ Methane emissions from this sector are characterized by significant temporal fluctuations,^7,8^ with sporadic, short-lived events of high emissions—known as super emitter events—accounting for a substantial portion of the total emissions.^9,10^ In this context, Continuous Monitoring Systems (CMS) that continuously measure methane concentrations are becoming increasingly vital for emission monitoring. They offer near real-time tracking capabilities essential for identifying both highly fluctuating emissions and transient super emitters.^11−13^ As such, CMS play a crucial role in detecting, localizing, and quantifying site emissions.^14−16^ Various types of CMS exist, each based on different measurement techniques. Scanning laser systems, for instance, measure integrated concentrations along an open path,^17^ whereas gas imaging systems generate 2D visualizations of concentration enhancements.^18^ Close-proximity continuous monitoring places intrinsically-safe sensors directly on equipment for detection and quantification.^19^ Additionally, point-in-space sensor networks measure concentrations at multiple fixed points at a distance from potential emission sources. This study primarily focuses on the latter category.

Point-in-space CMS sensor placement, the strategy of positioning multiple fixed point sensors on an oil and gas site, significantly affects the efficiency of methane emissions detection, especially when considering a limited sensor budget.^20^ Sensor placement optimization has been extensively studied in environmental monitoring applications such as air pollutant monitoring,^21,22^ water contamination monitoring,^23−27^ temperature monitoring,^28,29^ and wind monitoring.^30,31^ The goal is to optimize the locations of a limited number of sensors to maximize objectives such as coverage, detection capability, and data quality for subsequent analyses. Until recently, sensor placement optimization for methane emission monitoring on oil and gas sites had not been extensively studied due to CMS being a relatively new technology. Other methane emission monitoring technologies, such as satellite,^32^ aerial monitoring systems,^33^ and handheld optical gas imaging (OGI) cameras,^34^ do not require sensor placement. These systems can either cover entire sites (e.g., satellite and aerial systems) or be moved to various locations on-site (e.g., OGI). In contrast, as a ground-based monitoring system, CMS involves sensors that cannot be easily moved, and a limited number of sensors cannot cover an entire site. Therefore, effective sensor placement optimization methods are essential for maximizing the efficiency of methane emissions detection.

In the realm of methane emission monitoring at oil and gas sites, Klise et al.^35^ developed a sensor placement optimization framework designed to enhance the detection capabilities of sensor networks. This framework begins by employing site-specific wind data and emission characteristics to create simulated emission scenarios, thereby closely approximating the actual conditions present at the site. Following this, the framework applies mixed-integer linear programming (MILP) to identify optimal sensor placements. Klise et al.^35^ showcased their methodology through a case study within a confined space of 100 x 100 x 10 meters, arranging potential sensor locations at intervals of 10 meters horizontally and 1 meter vertically, culminating in a search space comprising 100,000 potential sensor locations. This complexity is further amplified by introducing three sensor categories with different detection threshold and cost, expanding the search space to 300,000 distinct configurations. Moreover, to refine the simulation of real-world conditions, 1,200 emission scenarios were generated, capturing a range of meteorological conditions and emission patterns. Building upon Klise et al.’s foundational work,^35^ Zi et al.^36^ introduced a novel approach by incorporating wind condition uncertainties and employing a distributionally robust optimization (DRO) strategy to bolster the sensor network’s detection robustness, particularly in worst-case scenarios. This methodology was evaluated using the same spatial domain as in Klise et al.’s study,^35^ demonstrating significant improvements in detection capabilities. Both studies leveraged MILP for optimization, showcasing its efficacy in smaller search spaces. However, practical applications such as reducing sensor spatial resolution to 1 meter on oil gas sites or extending the analysis to larger regions reveal the limitations of MILP due to its computational demands, which escalate exponentially with the increase in decision variables — in this context, the potential sensor locations. Although advanced computational strategies like branch-and-bound^37^ or branch-and-cut^38^ can alleviate some computational burdens, the nature of MILP algorithm means that solving them remains NP-hard in the most challenging scenarios.

In the present study, we endeavor to expand the search space for potential sensor locations and advocate for the utilization of genetic algorithms (GAs)^39^ to overcome the computational challenges encountered with MILP. GAs, distinguished by their emulation of natural selection processes, demonstrate high efficiency in navigating extensive and intricate solution spaces. Through the application of evolutionary mechanisms such as selection, crossover, and mutation, GAs are posited to achieve near-optimal solutions with greater rapidity and reduced computational burden compared to conventional MILP methods, particularly in instances where exact optimization proves to be computationally prohibitive. GAs have been widely used in sensor placement optimization across various applications, including structural health monitoring^40^ and seismic building monitoring.^41^ While GAs do not guarantee finding the exact optimal solution, their high efficiency in finding a sub-optimal solution that is very close to the optimum makes them well-suited for applications in methane emissions detection on oil and gas sites, where approximations in the modeled emissions make an exact solution superfluous. Within this context, we conceptualize the sensor placement as a multi-objective optimization problem guided by the principles of Pareto optimization^42^ which entails the simultaneous optimization of multiple conflicting objectives. Specifically, our objective is twofold: first, to identify a subset of sensor locations that optimizes detection capabilities, and second, to ensure that the number of selected sensors remains within a predetermined number. To address this dual-objective optimization problem, we employ the method developed by Qian et al.^43^ which leverages GAs to facilitate an effective solution search.

Compared to the work of Klise et al.^35^ and Zi et al.,^36^ we use a much higher spatial resolution to define possible sensor locations resulting in finding a sensor placement with higher detection efficiency. Also, we utilize all available wind data for a site with a resolution of 1 min to achieve a more precise approximation of the actual meteorological conditions at the site. Moreover, we simulate methane emissions with the Gaussian puff model rather than the Gaussian plume model owing to its superior accuracy in capturing the dynamics of atmospheric dispersion by allowing for variable wind conditions. As a result of the aforementioned enhancements, our sensor placement optimization algorithm delivers solutions of increased accuracy and granularity, thereby offering substantial benefits for practical applications. As such our algorithm offers an effective solution for optimal sensor placement which can serve as an open-source reference tool for point-in-space CMS technology vendors and advance the quest towards rapid elimination of methane emissions on oil and gas sites.

## Methods

In this study, we introduce a data-driven algorithm designed to optimize the placement of point-in-space CMS sensors. The algorithm is modular and a visual summary of the algorithm is provided in Section S1 in the Supporting Information (SI) document. The algorithm requires three critical inputs: site-specific wind patterns, emission characteristics, and a predefined number of sensors that can be placed (referred to as sensor budget). Utilizing these inputs, our algorithm calculates the optimal sensor arrangement for a given number of sensors, aiming to maximize emission detection efficiency, defined as the frequency or likelihood with which at least one sensor in the system detects an emission. Detection efficiency, the objective to be optimized in the sensor placement problem, can also be defined using other metrics such as time to first detection or detection fraction based on total time or amount of emissions. Due to the flexibility of GAs, the objective function can be adjusted accordingly without altering the overall framework. The workflow of the algorithm is structured into five distinct steps:

1. Generation of numerous emission scenarios that reflect the site’s actual emission characteristics and wind conditions.
2. Specification of possible sensor locations based on site geometry and operational guidelines if any.
3. Simulation of methane concentrations at all possible sensor locations for each emission scenario.
4. Evaluation of detection for each sensor location and emission scenario pair.
5. Optimization of sensor placements that ensure maximum detection across all scenarios under a given number of sensors to place.

In the following subsections, we first outline the experimental data utilized to showcase our algorithm. We then detail each step by first providing a general description and then explaining the experimental approach used in this study. It is important to reiterate that our algorithm is highly modular and that the components demonstrated in our experimental study, such as the wind and emission distribution estimators and the atmospheric transport model, can be readily replaced.

### Data Description

**Figure 1:**
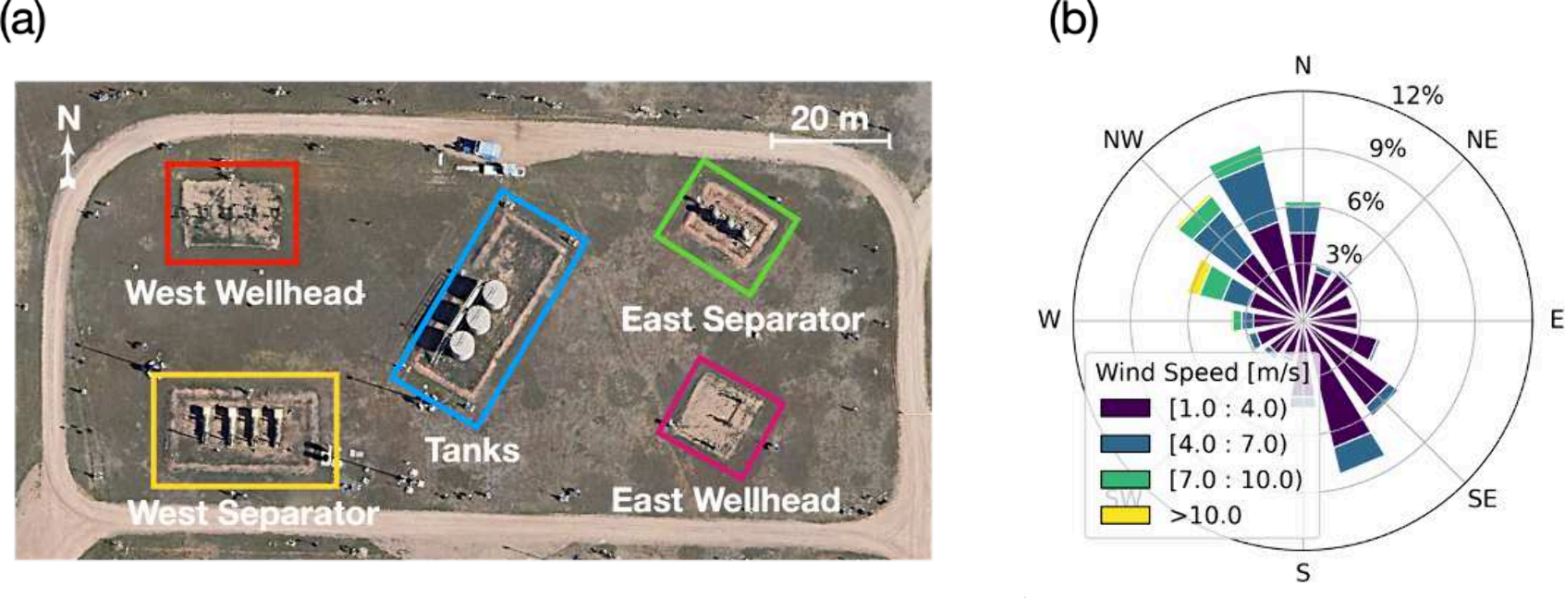
Experimental setup used to demonstrate the sensor placement optimization algorithm. (a) Satellite imagery of the Methane Emissions Technology Evaluation Center (METEC) site. Potential emission source locations are marked with colored boxes. (b) Distribution of wind data during the Advancing Development of Emissions Detection (ADED) experiment period from January 28 to May 16, 2022.

To validate the effectiveness of our proposed algorithm, we employ wind data acquired from the 2022 Advancing Development of Emissions Detection (ADED) campaign^44^ conducted at the Methane Emissions Technology Evaluation Center (METEC), which was operational from January 28 to May 16, 2022. Due to data availability and for demonstration purposes, we used a period of less than four months of wind data. In practice, a full year of wind data is recommended to accurately represent the actual meteorological conditions, including seasonal variations, at the site. This is implemented in the case study of a real oil and gas site as detailed in the final section of this manuscript. The configuration of the METEC site is illustrated in Figure 1a, where five distinct groups of equipment are outlined, each representing a potential emission source. Wind speed and direction were continuously recorded by three on-site anemometers throughout this period, with data captured at 1 min intervals. For the purposes of our analysis, we assumed wind conditions to be homogeneous across the site, thereby aggregating the data from the three anemometers to compute mean wind speed and direction. Note that the calculation of wind direction used the circular mean, appropriately addressing the circular nature of directional data. Figure 1b shows the wind rose that describes the wind distribution throughout the period.

### Step 1. Generation of Emission Scenarios

In accordance with the definition in Klise et al.,^35^ an emission scenario is determined by a combination of wind conditions and emission characteristics during a period of time. To generate emission scenarios, two probability distribution functions are constructed: a joint distribution *f*_*e*_ of emission source attributes including locations **x**_0_ (encompassing both horizontal and vertical coordinates), durations τ, and rates *q*, and a joint distribution *f*_*w*_ of wind speed ws(*t*) and wind direction wd(*t*), *t* ∈ [0, τ]. The wind distribution can be inferred from historical wind data recorded on or in the vicinity of the site. If observed wind data is unavailable, simulated data from models such as the High-Resolution Rapid Refresh (HRRR)^45^ can be utilized. The emission distribution can be obtained from operational logs and/or based on expertise from operators. It is important to note that emission rate characteristics may vary throughout the operational lifecycle,^46^ making it a key factor to consider when establishing equipment-level emission rate distributions. Due to the flexibility of the algorithm, operators can periodically re-run the algorithm when emission rate distributions change significantly, adjusting sensor placement as needed. Alternatively, they may incorporate anticipated future distributions into the initial emission scenarios to determine an optimized long-term placement. Practically, we can decompose the joint distribution *f*_*e*_ using conditional distributions, i.e., *f*_*e*_(**x**_0_, τ, *q*) = *f*(τ, *q*|**x**_0_) × *f*(**x**_0_). That is, each potential source, based on its functionality, may have its own emission pattern. The two distributions *f*_*w*_ and *f*_*e*_ are deemed independent, which we believe to be a reasonable assumption since the wind conditions should not affect the source attributes of an emission event and vice versa. Nevertheless, it is acknowledged that correlations may arise via confounding temporal factors. For instance, a certain type of emission may tend to occur at a specific hour of the day or in a specific season when the wind might have a specific pattern. In cases where prior knowledge pertaining to such relationships is available, it can be readily incorporated into the distributions facilitating a more nuanced modeling approach. Then, emission scenarios, which are defined by a tuple of (**x**_0_, *q*, τ, ws(t), wd(t), *t* ∈ [0, τ]), are generated by randomly sampling from *f*_*e*_ and *f*_*w*_.

In this case study, we consider single-source emissions—where only one source is active at a time—as our goal is to maximize detection efficiency. Thus, achieving successful detection of single-source emissions should inherently guarantee success in detecting multiple-source emissions. We note that the more challenging task of optimizing sensor placement to maximize information for emission localization and quantification is being explored in future work. For demonstration purposes, we fix the emission duration for each emission event to be 1 hour and assume all five equipment groups have equal likelihood to be an emission source. Additionally, we prescribe three possible emission rates, [1, 5, 10] kg/h to each of the potential emission sources with equal probability. This constructs a simple uniform distribution, i.e., *P*(**x**_0,*i*_, *q*_*j*_) = 1/15, *i* = 1, 2, ..., 5, *j* = 1, 2, 3. It’s important to mention that the fixed duration value, emission rates and probabilities we used in this experiment are only for demonstration purpose. In practice, a realistic distribution should be derived based on actual site-specific data and practitioners’ insights, and an example of this guidance is provided in the case study at the end of this paper. For wind data, we approximate the true wind distribution *f*_*w*_ by using 1 hour non-overlapping segments from the historical wind data throughout the entire experiment period to reflect the actual meteorological conditions on the site within the experiment period. In practice, a full year of wind data is desired to comprehensively represent the actual meteorological conditions, including seasonal patterns, which is implemented in the case study for a real oil and gas site. Finally, this yields a total number of 38,685 emission scenarios which represent the actual emission conditions on the site during the experiment period.

### Step 2. Specification of Possible Sensor Locations

The determination of possible sensor locations is contingent upon the desired spatial search grid resolution as well as the geometry of the site. Specifically, areas occupied by equipment or driveways must be excluded from the search space. Furthermore, regions restricted by operational guidelines also necessitate exclusion. It is not necessary to predefine locations where the plume cannot reach, as these locations will be automatically excluded in the subsequent optimization step. In summary, information regarding the search grid resolution sought by the site operator and any pertinent restrictions is essential for the specification of possible sensor locations.

**Figure 2:**
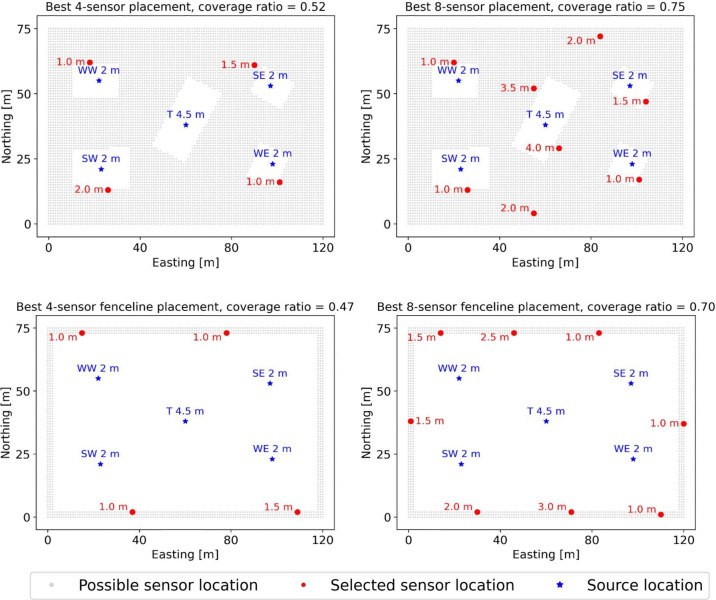
Optimal placements for four and eight sensors as determined by the Pareto Optimization with Recombination for Subset Selection (PORSS) algorithm. The top row shows the optimal sensor placements across the entire site, and the bottom row shows the optimal sensor placements constrained along the fence line of the site. Gray dots indicate possible sensor locations, while blue stars represent potential emission source locations, with their heights indicated by the adjacent numbers. Red dots denote the sensors selected by the algorithm, with their corresponding heights also indicated by the adjacent numbers. The optimal placement of four sensors across the site and along the fence line achieves detection coverage of 52% and 47%, respectively. The optimal placement of eight sensors across the site and along the fence line achieves detection coverage of 75% and 70%, respectively.

In this case study, we first set possible sensor locations by creating a 3D grid over the entire site. The resolution for the two horizontal directions (Northing and Easting) is 1 m while the resolution for the vertical direction is 0.5 m. The dimensions of the METEC site are approximately 125 meters in Easting and 75 meters in Northing. The vertical range is broadly set from 1 to 10 m to demonstrate the scalability of our algorithm. In practical applications, a user-specified narrower range at lower heights may be considered based on safety or installation feasibility. Subsequently, we excluded all points within the grid that were occluded by any of the five equipment groups present on the site. This exclusion process resulted in a total of 146,110 viable sensor locations, forming the search space for the subsequent optimization analysis. Figure 2 shows the possible sensor locations as well as the potential emission sources on the METEC site. The gap areas correspond to the spaces occluded by equipment and hence devoid of possible sensor locations.

### Step 3. Simulation of Methane Concentrations

In this step, we simulate methane concentrations for each emission scenario generated in Step 1 at all potential sensor locations specified in Step 2, using an atmospheric transport model. This step is summarized by eq 1:
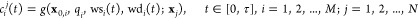
1Here, *c*_*i*_^*j*^ represents the simulated methane concentration time series for emission scenario *i* at sensor location *j*; *M* and *N* denote the total numbers of emission scenarios and potential sensor locations, respectively; *g* is the atmospheric transport model. We will use these simulated methane concentrations to assess detection capabilities at each sensor location for each emission scenario.

In our experiment, the simulations were conducted using the Gaussian puff model which is more accurate than the Gaussian plume model as it accounts for the wind dynamics within the simulation period (see Jia et al.^47^ for details). All the parameters used in the Gaussian puff simulations are listed in Section S2 of the SI. We note that to address limitations of the Gaussian puff model, such as its inability to account for physical obstructions, more sophisticated atmospheric transport models can be straightforwardly integrated. Finally, there are a total of 38, 685 × 146, 110 = 5, 652, 265, 350 1 h simulated concentration time series, each with 1 min resolution. Memory issues arise when dealing with such large data. To address them, we utilize memory-mapping files provided in NumPy^48^ to store the data.

### Step 4. Evaluation of Detection

In this step, we establish detection of every sensor location and emission scenario pair based on the simulated methane concentrations *c*_*i*_^*j*^ obtained in the previous step. We then combine all the detection results into a single detection matrix which serves as the input to the final optimization problem.

We determine detection by applying two thresholds to the simulated methane concentrations: an amplitude threshold, *A*, to identify significant high values and a temporal threshold, *B*, to ensure the persistency of the high readings. Therefore, a successful detection is defined to be elevated concentrations above *A* ppm for more than *B%* of the total time steps in a simulated concentration time series as shown in eq 2.
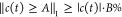
2where *c*(*t*) is the simulated methane concentration time series (or vector); ∥ ·∥_1_ is the *L*_1_ norm of a vector and |·| is the length of a vector. The value of *A* will typically be set as a function of the sensor quality, with lower quality sensors requiring a higher threshold to distinguish true spikes from the noisy background which consists of ambient atmospheric concentrations and sensor noise. Since we use simulated concentrations in this step, representing concentration enhancements in the observed data, the detection threshold *A* should be based on the background variations in the observed data to ensure that the signal corresponding to true emissions stands out. Specifically, *A* is set to be the difference between the upper bound and the lower bound of the background concentration, ensuring that any concentration enhancement above *A* is guaranteed not to be drowned out by the background. The value of *B* dependents on the tolerance for false positives. In practice, sporadic spikes may correspond to sensor malfunctions or error from raw data processing rather than actual emissions. We need a metric to evaluate the persistence of the high values defined using the amplitude threshold *A*. In this study, we use an amplitude threshold of *A* = 0.5 ppm and *B* = 20% for the main analysis. An example of a successful detection is illustrated in Section S3 of the SI. We also analyzed how different sensor types may affect the detection efficiency by using *A* = 5.0 ppm and *B* = 20% to generate results corresponding to a lower quality sensor. Our algorithm also includes an additional metric for evaluating persistence: the number of consecutive high-value points, which can be set based on user preference. We included a brief analysis of different choices in Section S4 of the SI. Note that, in this study, we focus on detection coverage, the proportion of emission events detected, rather than the time to detection—an alternative commonly used metric for assessing detection efficiency.^20^ The time to detection metric can be incorporated by adjusting the definition of detection in this step, and will be explored in future work.

Next, we synthesize all detection results to construct a detection matrix, denoted as *D*, whose rows correspond to sensor locations and columns to emission scenarios. The entries of the matrix are binary; zero indicates a non-detection for a specific scenario at that sensor location, and one indicates a detection, see eq 3.
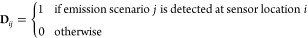
3

### Step 5. Optimization of Sensor Placement

In the final step, we find the optimal sensor placement under a given number of sensors to place. Using the detection matrix *D* constructed in the previous step, we can model the optimization problem as a best subset selection problem. Recall that the rows of the detection matrix correspond to possible sensor locations and columns to emission scenarios. Given the number of sensors *k*, selecting *k* sensors translates to selecting *k* rows from the detection matrix. In the resulting sub-matrix *D*_sub_^(*k*)^, we assess the column-wise sums which indicate the number of sensors that detect the corresponding emission scenario. The number of non-zero column-wise sums is the total number of detected emission scenarios, which is called detection coverage. The objective is to find the optimal selection of *k* rows from this matrix to maximize the detection coverage.

The next step is to apply Pareto optimization to the best subset selection problem we have formulated. Pareto optimization has been developed to handle situations with conflicting objectives. In our case, we want to find a subset of rows from the detection matrix to maximize the detection coverage, while minimizing the size of the subset, that is, using fewer sensors if possible. The Pareto optimization problem is formulated by following the set up used in Qian et al.^43^
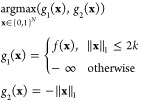
4

Here, *N* is the number of rows of the detection matrix, **x** is a binary vector of length *N* and **x**_*i*_ = 1 if the *i*th row of the detection matrix is selected; *f* is the detection coverage and ∥**x**∥_1_ is the *L*_1_ norm of the binary vector which is the number of selected rows. Note that for solutions which unduly exceed the number of sensors to place (by two times in this setting), the corresponding objective value in *g*_1_ is set to be negative infinity to make the subsequent searching process more efficient.

For large-scale optimization problems, such as those involving matrices with dimension in the order of hundreds of thousands, traditional approaches like exhaustive search or standard MILP algorithms are impractical due to their computational cost. Instead, here we use GAs, a type of randomized algorithms to efficiently search for a solution that is close to the optimal one. By conducting selection, mutation, and combination operations inspired by natural selection, the solutions evolve to an optimal or near optimal state in an efficient way. Specifically, we use the Pareto Optimization with Recombination for Subset Selection (PORSS) proposed by Qian et al.^43^ The pseudo-code for the algorithm is presented in Section S5 in the SI and the full code is available at the GitHub repository provided at the end of this paper. The algorithm accepts three primary inputs: a detection matrix **D**, the number of sensors *k*, and an objective function *g*_1_ which is the detection coverage in this case. The iterative nature of the algorithm is governed by the parameter *I*, specifying the total number of iterations to execute. The desired output is an optimized subset comprising no more than *k* rows from the detection matrix **D** that maximizes the objective function *g*_1_. Given the monotonic property of *g*_1_, which ensures that the objective value of the original set is always greater than or equal to that of any of its subsets, the output will consist precisely of *k* rows.

We start with a null vector **x** of length *N*, the total number of possible sensor locations, initializing a solution population set *P* with this vector. The iteration counter *i* is set to zero to track the progression of the algorithm.

The core of the algorithm is an iterative loop that continues until either the iteration counter *i* reaches the predefined limit *I* or the prescribed early stop condition is satisfied. Within each iteration, the following steps are undertaken:

1. Selection: A pair of vectors, **x**, **y**, are randomly chosen from the solution population set *P*.
2. Recombination: The selected vectors undergo a recombination process to produce offspring vectors **x′**, **y′**. Specifically, a cut point is randomly selected to split each of **x**, **y** into two segments: (**x**_1_, **x**_2_), (**y**_1_, **y**_2_). Then, **x**_1_ is combined with **y**_2_, and **y**_1_ is combined with **x**_2_ to produce the offspring vectors **x′** and **y′**.
3. Mutation: A bit-wise mutation is applied to the offspring **x′** and **y′**, resulting in new vectors **x″**, **y″**. Specifically, a subset of points is randomly selected, and the values at these points are swapped between 0 and 1 to produce the new vectors **x″**, **y″**.
4. Survival of the Fittest: For each of **x″** and **y″**, the algorithm assesses whether it represents an improvement over the existing solutions in *P*. Only vectors not dominated by any member of *P* are retained. Here, we define a solution **x** dominating another solution **x′**, i.e., **x** ≻ **x′** if *g*_1_(**x**) ≥ *g*_1_(**x′**) and *g*_2_(**x**) ≥ *g*_2_(**x′**) **and** either *g*_1_(**x**) > *g*_1_(**x′**) or *g*_2_(**x**) > *g*_2_(**x′**).
5. Early Termination: At the end of each iteration, an early stop condition is evaluated to determine if the optimization can be concluded ahead of schedule.

Upon reaching the termination criterion, the algorithm concludes by selecting from *P* the solution **x** that adheres to the number of sensors constraint *k* and maximizes the objective function *g*_1_.

The proposed algorithm demonstrates a robust capability for optimizing complex subset selection problems. Through the iterative process of selection, recombination, mutation, and survival of the fittest, the algorithm converges towards an optimal or near-optimal solution. The inclusion of an early termination condition enhances computational efficiency, enabling the algorithm to halt upon sufficient convergence, thereby saving computational resources.

To address the inherent stochastic nature of GAs which can result in diverse outcomes due to their random selection and mutation processes, we have implemented a strategy that involves running multiple independent GAs in parallel. By conducting these algorithms separately, we can harness the variability in their search patterns, thereby mitigating the risk of converging to local optima that might be mistaken for global solutions. Upon completion of these independent runs, we aggregate their results, systematically comparing and combining the distinct solutions to identify the most optimal outcomes. This approach not only leverages the exploratory strengths of GAs but also significantly enhances the robustness of the solution set, ensuring that the best possible solutions are identified amid randomness. Details on computational resources and runtime analysis are provided in Section S6 of the SI.

To evaluate the performance of our algorithm, we generated test data with known ground truth. These test data were designed to present more challenging scenarios than the original problem in this study, and our algorithm successfully identified the true solution in all cases. Detailed descriptions and results of the tests are provided in Section S7 of the SI.

## Results

We first present the optimal placement of sensors across the METEC site, considering a sensor budget ranging from 1 to 12 sensors. For each number, we conducted 20 independent optimization trials and aggregated the results to identify the most effective sensor configuration. We consider two sets of candidate sensor locations: one covering the entire site domain, excluding areas occupied by equipment, to demonstrate the scalability of our algorithm and its benefits over fence line placements; and one focused specifically on fence line placement, which is currently the industry standard. Figure 2 showcases exemplary configurations for the optimal placement of four and eight sensors, with additional configurations for varying numbers of sensors detailed in Section S8 of the SI. The sensors’ horizontal positions are marked with red dots, while their heights are denoted by corresponding numbers. The top row shows the optimal sensor placements by searching from all possible sensor locations specified in Step 2 across the entire site. The detection coverage achieved by these configurations is 0.52 and 0.75 for the four-sensor and eight-sensor systems, respectively, indicating that the systems could detect 52% and 75% of simulated emission scenarios, respectively. Not surprisingly, the arrangement of the best sensor placement is clearly informed by the predominant wind directions from the northwest and southeast (see Figure 1b). Additionally, the sensor heights are highly correlated with the heights of the emission sources. For fence line sensor placement optimization, we limited potential sensor locations to a narrow zone along the site boundary, specifically within a 2-meter buffer area. The bottom row shows the optimal configurations for placing four and eight sensors, as determined by our algorithm. Again, the arrangement of the optimal sensor placements is informed by the predominant wind directions. Specifically, rather than being uniformly distributed along the perimeter, the sensors are shifted towards the northwest and southeast direction, corresponding to the predominant wind directions over the experiment period. The four sensor locations in the best four-sensor solution are approximately retained in the best eight-sensor solution, but their heights are adjusted to synergize with the newly added sensors, enhancing detection efficiency.

**Figure 3:**
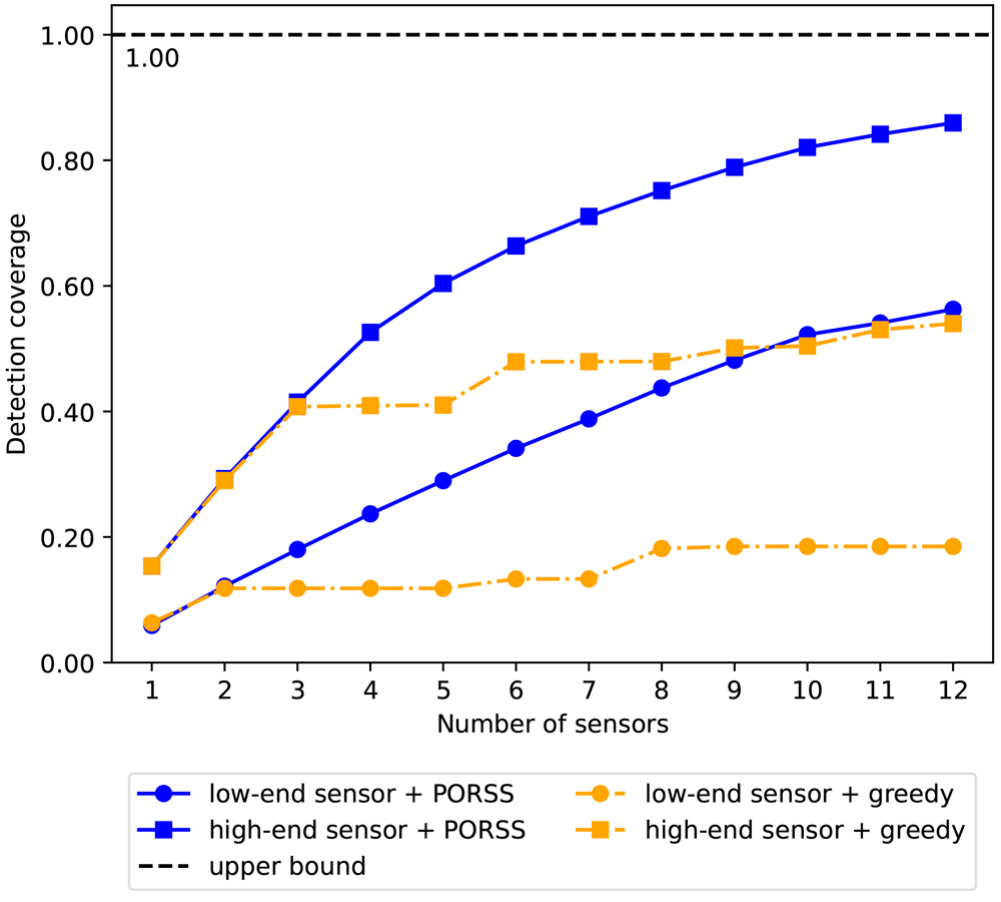
Detection coverage ratios achieved through optimal sensor placement across various numbers of sensors. Blue solid lines denote solutions derived from the PORSS algorithm, while orange dash-dot lines represent outcomes from the greedy search method. Circles indicate solutions achieved under a detection amplitude threshold of 5.0 ppm, representing the sensitivity of low-end sensors. Squares denote solutions achieved under a detection amplitude threshold of 0.5 ppm, representing the sensitivity of high-end sensors. The black dashed line illustrates the theoretical maximum coverage ratio, achievable by distributing sensors across all possible locations.

Figure 3 illustrates the detection coverage ratio, the proportion of detected emission scenarios out of the total emission scenarios generated in Step 1, achieved with the optimal sensor placement for each number of sensors. The blue lines show the coverage ratio associated with the optimal sensor placements derived by the PORSS algorithm in this setting. The observed trend indicates diminishing returns in terms of detection coverage with increasing number of sensors. This type of analysis enables us to determine the minimum number of sensors required for a desired detection coverage. Additionally, we compared the results under two different detection amplitude thresholds: 0.5 and 5 ppm representing high- and low-end sensors, respectively. Low-end sensors generally exhibit larger background variation compared to high-end sensors and hence require higher detection thresholds.^49^ The analysis shows that to achieve a specific detection coverage ratio, more low-end sensors are needed. For example, to achieve a detection coverage ratio of 0.50, four high-end sensors or nine low-end sensors are required. In practice, operators can use this analysis to decide on the sensor type and the corresponding number of sensors. A basic comparison of detection coverage between CMS and other measurement technologies, including optical gas imaging (OGI), aerial, and satellite, is provided in Section S9 of the SI. The comparison reveals that the 4-sensor CMS achieves significantly higher detection coverage than the other methods. Cardoso-Saldaña^50^ provides a more in-depth analysis of emission reduction efficiency across various monitoring technologies.

The effectiveness of the PORSS algorithm was further evaluated by benchmarking it against a greedy search method which served as the baseline for comparison. In the greedy search approach, the best current row is iteratively chosen based on a continually updated detection matrix. This matrix evolves by excluding previously selected rows and any columns containing at least one value of '1' across these rows. As depicted in Figure 3, the solutions derived from the PORSS algorithm represented by the blue lines consistently outperform those obtained through the greedy search method represented by orange lines for both low- and high-end sensors. Notably, for the single-sensor (best-1) configuration, the solution identified by the greedy search is considered the ground truth, which PORSS successfully matched. This outcome underscores the PORSS algorithm’s ability to find optimal solutions.

## Case Study for a Prototypical Midstream Oil and Gas Site

In this section, we present a case study demonstrating our sensor placement algorithm in a real-world scenario at the request of a site operator. The study illustrates how operator knowledge and site constraints can be incorporated into our optimization scheme for an actual midstream oil and gas site. The site operator and site locations are anonymized for confidentiality.

This site initially had nine industry-standard sensors placed on the perimeter fence line, following the vendors recommendation. The operator wanted to compare this setup to a new configuration using four higher-quality sensors. Given that precise placement becomes more critical with fewer sensors, our framework was used to determine the optimal locations for the new sensors under the operator constraint of restricting placement to the fence line at a 2-meter height to facilitate installation and minimize operational interference.

Our optimization framework was configured as follows:

### Wind Data

**Figure 4:**
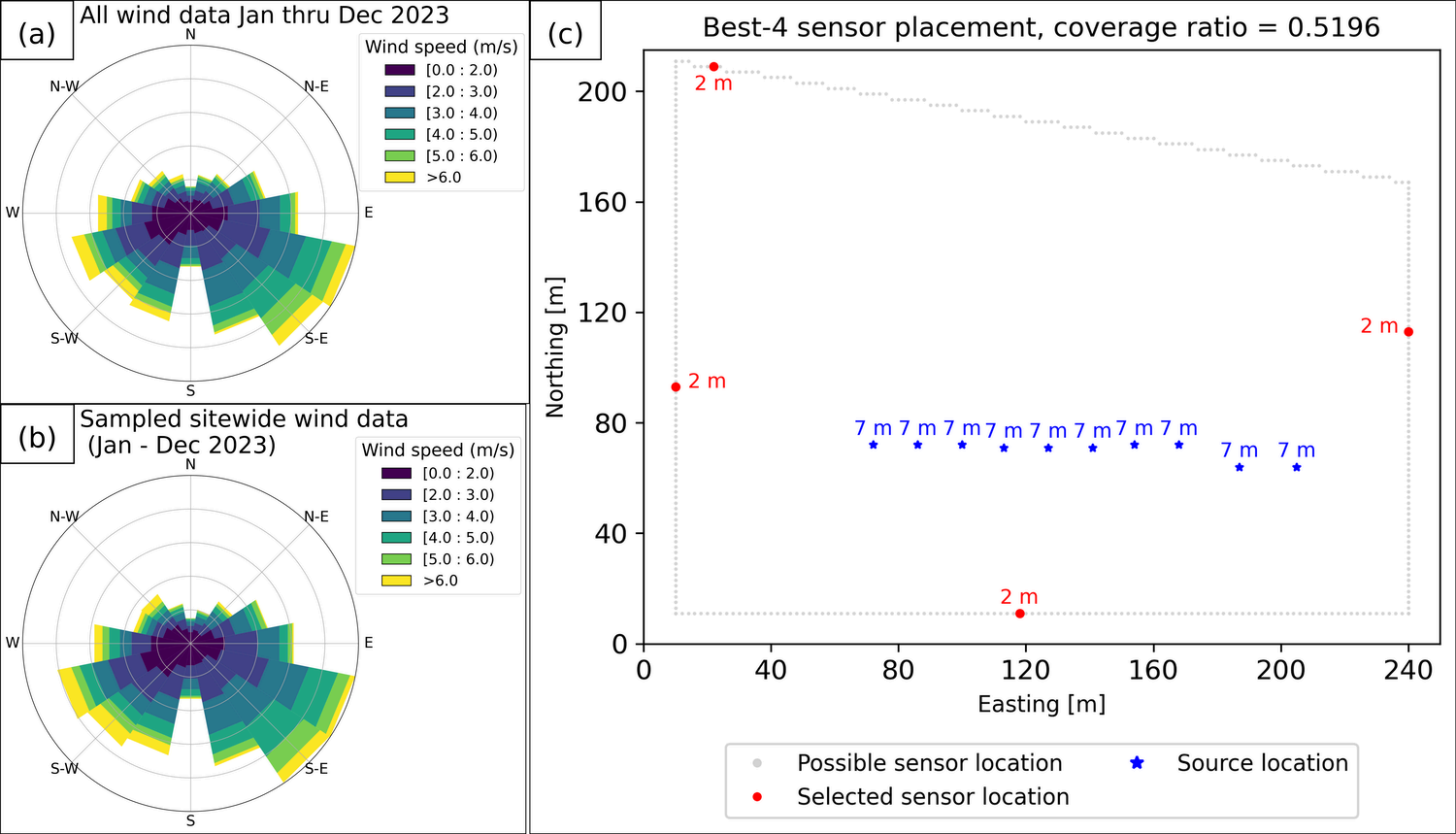
Subplot (a) shows the distribution of wind data recorded over a full year for this site. Subplot (b) shows the distribution of the subset of wind data used within our optimization framework. Subplot (c) shows the optimal fence line placement for four sensors on this site.

Wind data was collected over a year at 1 min intervals from two anemometers co-located with the existing sensors. To create a single time series for our framework, we averaged the wind speed and direction (x and y component wise) when both anemometers had data, using the available value when one was NA. Instances with both NA (0.684% of the data) remained NA. We then randomly selected 1,000 nonconsecutive hours of gap-free data, with the sampled distribution closely matching the full year’s data (see Figure 4a, b).

### Emission Scenarios

Operator guidance indicated that the 10 compressors on the site were the primary sources of fugitive emissions, and overflight data had suggested that emissions of 5 kg/h were typical for each compressor. We simulated emissions using these values across the 1,000 hourly wind data samples for each compressor, generating 1,000 scenarios per compressor and 10,000 total for all sources combined, allowing us to find the overall optimal placement.

### Simulation, Detection, and Optimization

All simulations used the Gaussian puff model with parameters listed in Section S2 of the SI. For each simulation, we recorded the candidate locations along the 2 m resolution site fence line where the simulated methane concentration exceeded 1 ppm for at least 1 min in accordance with a conservative estimate of sensor sensitivity provided by the sensor vendor. We first determined optimal sensor placements for each compressor individually (see Figure S9 in the SI) then combined the results to create a single detection matrix **D** as described in Step 4. The PORSS algorithm (see Step 5) was then used to find the optimal sensor locations. Lastly, we verified our solution’s stability by comparing it against 10 million random samples of 4 sensor locations along the fence line, imposing a 30 m minimum distance between sensors. The PORSS algorithm found the optimal solution in 210 seconds, about 30 times faster than evaluating the random samples which took 6,177 seconds, with nearly identical results but a slightly higher coverage ratio for the PORSS algorithm (see Figure S10 in SI).

The site operators have installed the four new sensors in the recommended locations and are actively recording data as of this paper’s publication. While a detailed comparison between these four advanced sensors and the nine existing ones is beyond this paper’s scope, this case study illustrates the practical implementation of our framework, integrating operator knowledge effectively.

## Conclusion

In this work, we introduced an open-source data-driven algorithm for optimizing point-in-space continuous monitoring systems (CMS) sensor placement on oil and gas sites. The demonstration example (METEC experiment) and the real oil and gas site case study outlined in this paper detail the workflow and highlight the efficiency and accuracy of our proposed framework. This algorithm provides site-specific solutions that are more accurate and reliable than traditional sensor placement strategies and those proposed in prior studies.^35,36^ Additionally, our algorithm’s analysis of the relationship between the number of sensors and detection efficiency is crucial for operators when making decisions during the planning phase.

Our approach significantly expands the problem scale compared to prior research in related studies by efficiently solving large-scale problems often encountered in practice. Further, the high modularity of our algorithm allows users to incorporate their own methods and models, such as wind and emission distribution estimators (in cases with limited or no existing wind data) or different atmospheric transport models. As such, the algorithm holds substantial practical value, particularly as the use of CMS becomes increasingly prevalent and critical in the quest to rapidly reduce methane emissions. While this paper focuses on optimizing sensor placement for methane emission detection efficiency, the algorithm can be extended to various other applications, involving emission localization and quantification, by simply changing the objective function in the optimization problem. This flexibility is due to the genetic algorithm’s ability to handle nonlinear objective functions, a capability not possible with the Mixed-Integer Linear Programming (MILP) approach adopted in prior studies.^35,36^

Future research directions include optimizing the combination of different types of continuous monitoring sensors, such as low-cost close-proximity sensors and point-in-space sensors, to simultaneously increase detection coverage and reduce costs. Additionally, implementing the genetic algorithm within a distributionally robust optimization framework^36^ can account for uncertainties inherent in emission scenarios.

## Data Availability

The code is available at https://github.com/Hammerling-Research-Group/placement.git.

## Supporting Information Available

Additional details about Gaussian puff simulation, sensitivity analysis for different detection thresholds, model runtime analysis, model validation on test data, and case study (PDF)
