# Supplementary material for: Optimizing Point-in-Space Continuous Monitoring System Sensor Placement on Oil and Gas Sites
Source: ACS Sustain Resour Manag. 2024 Dec 18;2(1):72–81. doi: 10.1021/acssusresmgt.4c00333 (PMC11770763; doi:10.1021/acssusresmgt.4c00333)
Supplement: Supplementary file 1 — rm4c00333_si_001.pdf [file rm4c00333_si_001.pdf]

Supporting Information for:

Optimizing point-in-space continuous monitoring systems sensor placement on oil and gas sites

Meng Jia[1], Troy Robert Sorensen[1], and Dorit Martina Hammerling[1,2]

[1]Department of Applied Mathematics and Statistics, Colorado School of Mines, Golden, Colorado 80401, United States
[2]Energy Emissions Modeling and Data Lab, The University of Texas at Austin, Austin, Texas 78712, United States

Email: mjia@mines.edu

Contents

S1 Visual Summary of the Sensor Placement Optimization Algorithm **2**

S2 Methane Concentration Simulations Using the Fast Gaussian Puff Implementation **3**

S3 Example of a Successful Detection under Given Thresholds **3**

S4 Sensitivity Analysis for Different Detection Thresholds **4**

S5 Pseudo-Code for the PORSS Algorithm **7**

S6 Computational Resources and Algorithm Runtime Evaluation **8**

S7 Performance Evaluation on Test Data **9**

S8 Optimal Sensor Placements under Different Number of Sensors **10**

S9 Comparison of Detection Coverage between Continuous Monitoring Systems and Other Monitoring Technologies **15**

S10 Additional Analysis from the Case Study for a Prototypical Midstream Oil and Gas Site **15**

S1 Visual Summary of the Sensor Placement Optimization Algorithm

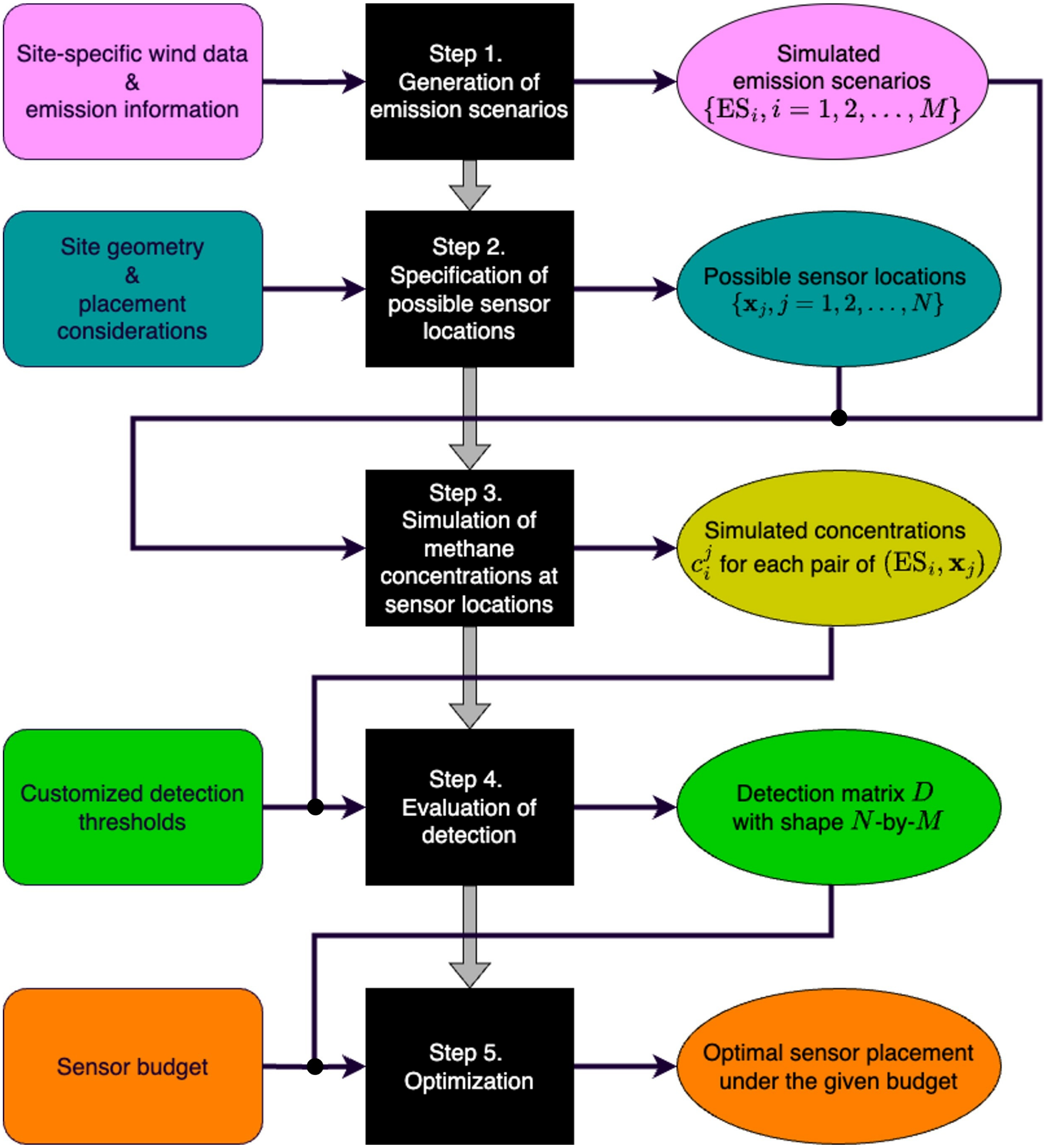

Figure S1: Summary flowchart for the sensor placement optimization proposed in this study. The five main steps are represented with black rectangles, and the inputs and outputs of each step are represented with colored rounded rectangles and ellipses, respectively.

S2 Methane Concentration Simulations Using the Fast Gaussian Puff Implementation

In this study, we utilized the fast Gaussian puff implementation proposed in [Jia et al., 2024] to simulate methane concentrations for each emission scenario and possible sensor location pair. The Gaussian puff model offers greater accuracy compared to the commonly used Gaussian plume model, as it accounts for wind variability by relaxing the steady-state assumption inherent to the Gaussian plume model. This fast Gaussian puff model employs dynamic thresholding to significantly reduce runtime, making it feasible for large-scale computations. Specifically, this study addresses a total of 5,652,265,350 emission scenario and sensor location pairs (38,685 emission scenarios $\times$ 146,110 sensor locations).

The fast Gaussian puff implementation uses two parameters to balance accuracy and computational speed: Δt and Δp. The parameter Δt affects the accuracy of individual puff simulations, while Δp influences the approximation of continuous emissions by discrete puffs. Following the guidelines from Section 4.2 in [Jia et al., 2024], we used $\Delta t = 1s$ and $\Delta p = 4s$ to achieve an optimal balance between simulation accuracy and computational cost. For further details and the associated code, refer to [Jia et al., 2024].

S3 Example of a Successful Detection under Given Thresholds

In Step 4 of the algorithm, detection for an emission scenario at a possible sensor location is determined by evaluating the corresponding simulated methane concentrations using two thresholds: an amplitude threshold A and a temporal threshold B. Figure S2 illustrates an example of a successful detection with the threshold values set at $A = 0.5$ ppm and $B = 20\%$, as used in the main analysis of this study.

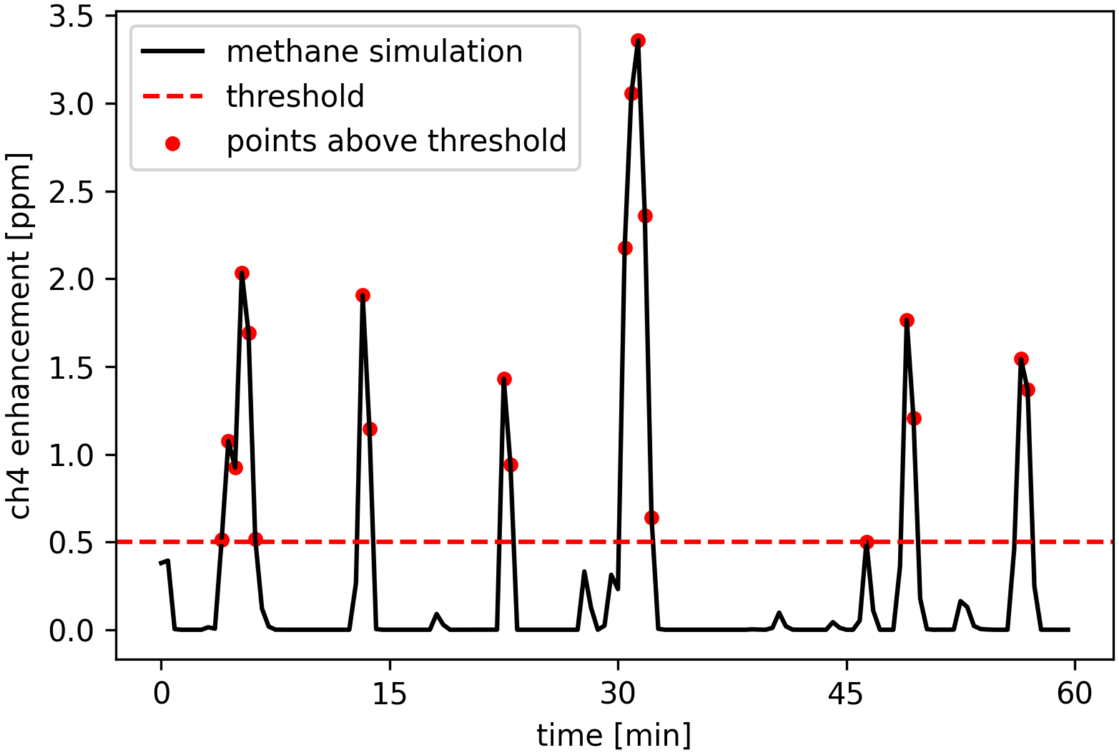

Figure S2: Illustration of the Gaussian puff simulation and subsequent detection evaluation using the proposed algorithm. The black line represents the time series of simulated methane concentration, while the red dashed line indicates the specified amplitude threshold required for detection. Points in the simulation surpassing this threshold are marked with red dots. Among the 60 data points analyzed, 20 (33%) exceed the amplitude threshold which is over the temporal threshold specified at 20%, qualifying this as a successful detection event.

S4 Sensitivity Analysis for Different Detection Thresholds

In this section, we perform a brief analysis using different detection threshold schemes. As described in the main text, two thresholds are used to determine detection: an amplitude threshold A in [ppm], which separates concentration enhancements from background levels, and a temporal threshold B, which characterizes the persistence of the concentration enhancement. Here, we investigate how the choice of these thresholds may affect the optimal sensor placements. For the amplitude threshold, we use the same values, 5.0 ppm and 0.5 ppm, as used in the comparison of low-end and high-end sensor types in the main text. For the persistence threshold, we investigate four methods: single-point, where a detection occurs if any single point of the simulated methane concentration exceeds A ppm; 20% aggregate points, as used in the main text; 3-consecutive points, where detection occurs if three consecutive points exceed A ppm; and 5-consecutive points. The optimal four-sensor placements derived by our algorithm under these different detection threshold schemes are shown in Figure S3. The rows correspond to different persistence thresholds as labeled on the left of each row, while the columns correspond to different amplitude threshold values A as labeled on the top of each column. We can observe that the optimal sensor placements are relatively robust for the high-end sensor type, as shown in the left column. For the low-end sensor type, the optimal sensor placements vary under different persistence thresholds. In practice, users need to be very careful in this case and choose the appropriate persistence threshold based on the acceptable level of false positive detections. This means that if the tolerance for false positives is low, a stricter

persistence threshold should be chosen to ensure the fidelity of detections. Figure S4 illustrates the relationship between the number of sensors and the corresponding detection coverage ratio achieved with the optimal sensor placement under different detection thresholds. We can see that for both amplitude thresholds, the persistence thresholds become increasingly stricter in the following order: single point, 3-consecutive points, 5-consecutive points, and 20% aggregate points.

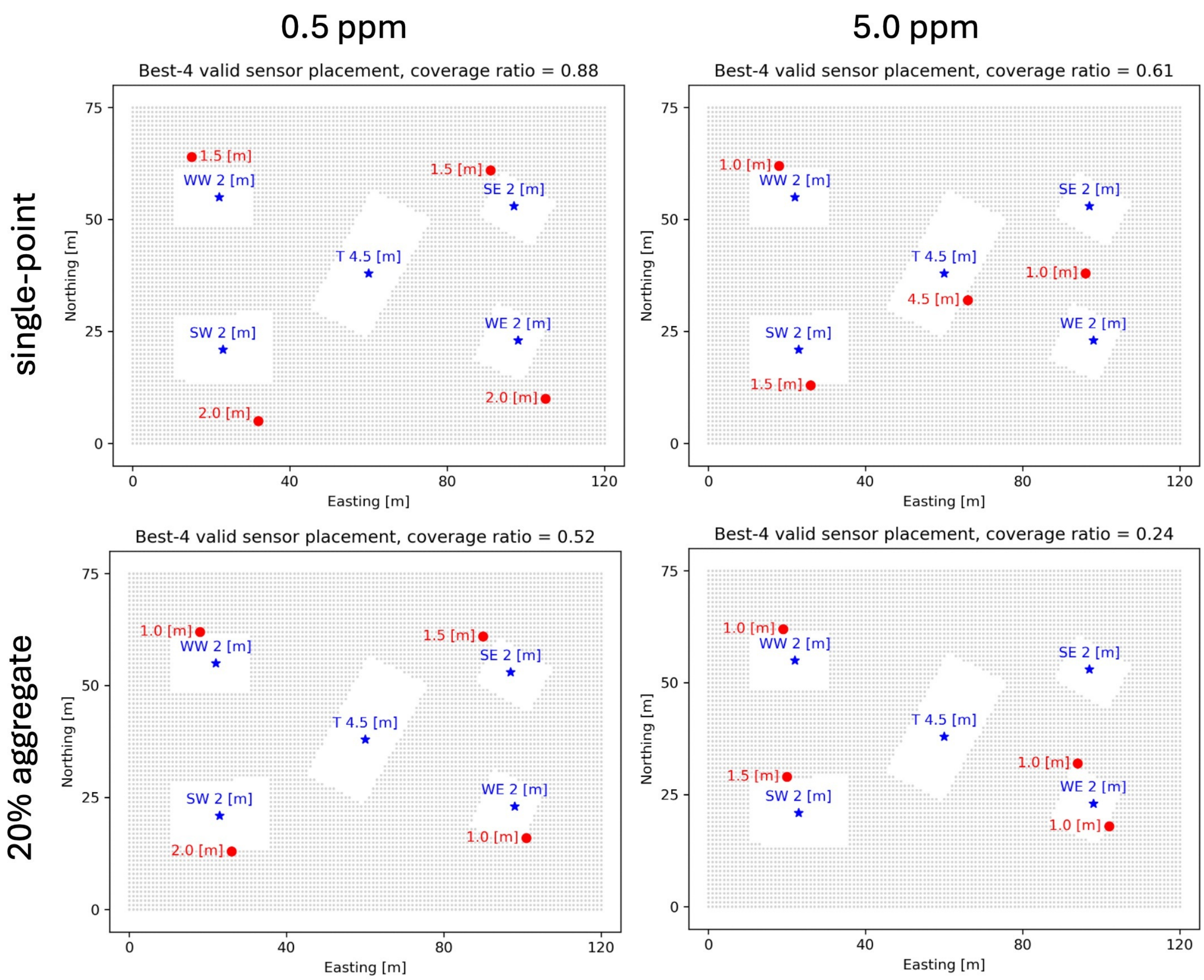

Figure S3: The optimal four-sensor placements derived by our algorithm under different detection threshold schemes. The rows correspond to different persistence thresholds: single-point (a detection occurs if any single point of the simulated methane concentration exceeds A), 20% aggregate points (as used in the main text), 3-consecutive points (a detection occurs if three consecutive points exceed A), and 5-consecutive points. The columns correspond to different amplitude threshold values (A) of 0.5 ppm and 5.0 ppm, representing high-end and low-end sensor types, respectively. The notations in each subplot are the same as in Figure 2 in the main text. (Continued on the next page.)

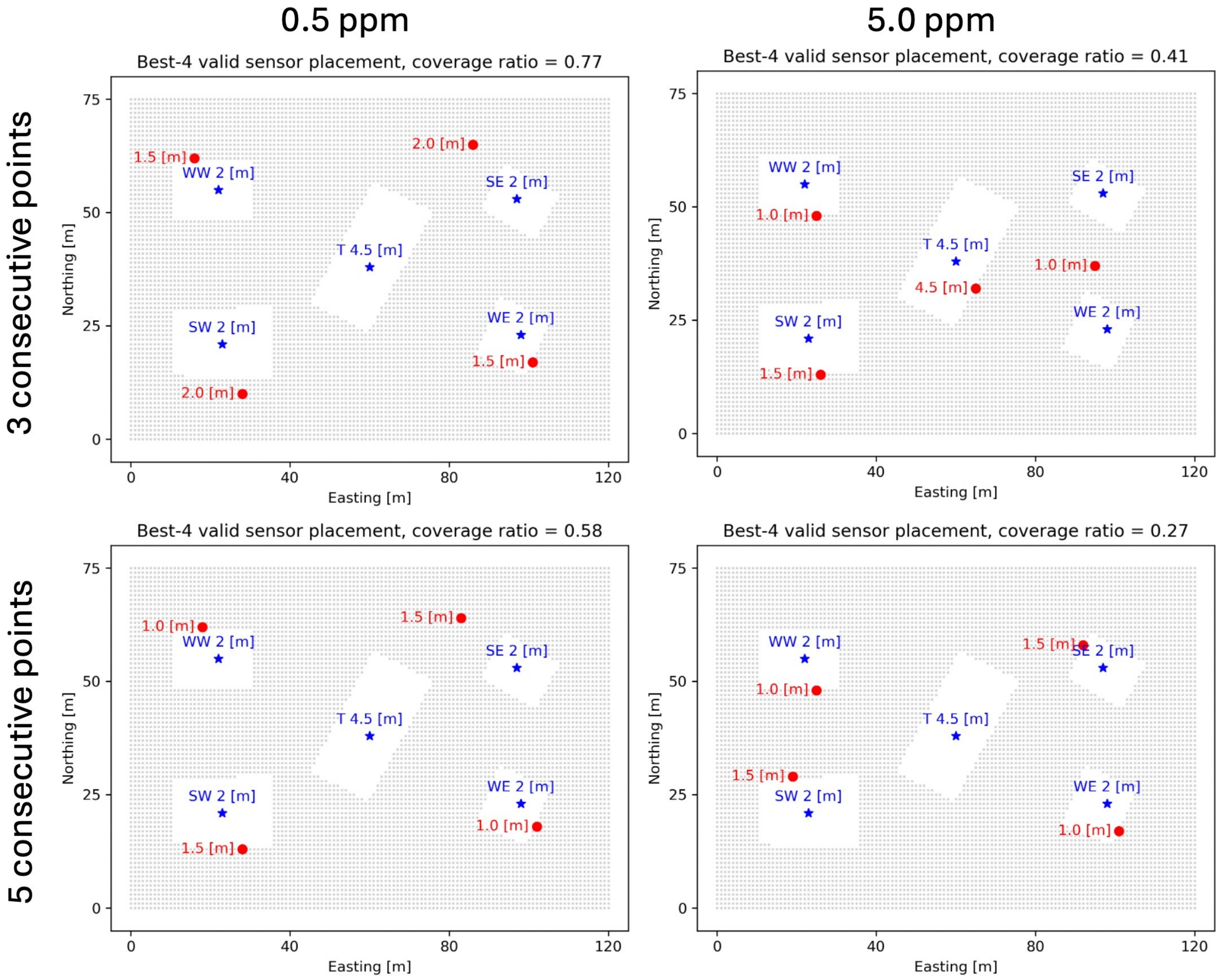

Figure S3: (Continuation of the previous page) The optimal four-sensor placements derived by our algorithm under different detection threshold schemes. The rows correspond to different persistence thresholds: single-point (a detection occurs if any single point of the simulated methane concentration exceeds A), 20% aggregate points (as used in the main text), 3-consecutive points (a detection occurs if three consecutive points exceed A), and 5-consecutive points. The columns correspond to different amplitude threshold values (A) of 0.5 ppm and 5.0 ppm, representing high-end and low-end sensor types, respectively. The notations in each subplot are the same as in Figure 2 in the main text.

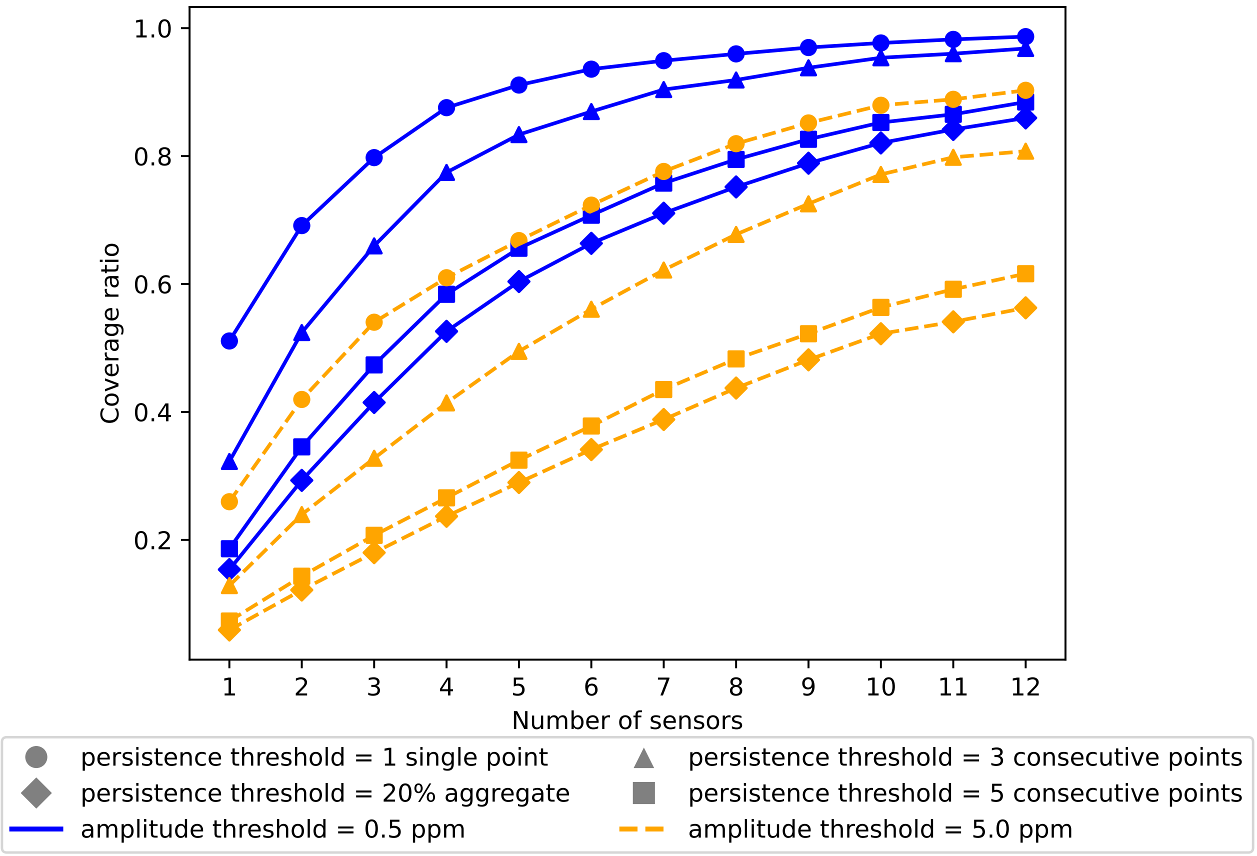

Figure S4: Detection coverage ratios achieved through optimal sensor placement across various numbers of sensors under different detection thresholds. The blue solid lines represent an amplitude threshold of 0.5 ppm, while the orange dashed lines represent an amplitude threshold of 5.0 ppm. The symbols correspond to different persistence thresholds: circles for single-point, diamonds for 20% aggregate points, triangles for 3-consecutive points, and squares for 5-consecutive points.

S5 Pseudo-Code for the PORSS Algorithm

Here, we present the pseudo-code for the PORSS algorithm used in Step 5 of our proposed sensor placement optimization framework.

Algorithm 1 PORSS Algorithm

Input: detection matrix $\mathbf{D}$; number of sensors k; detection coverage function g_1;
Parameters: the number I of iterations
Output: selected k rows of $\mathbf{D}$
Let $\mathbf{x} = \{0\}^N$, $P = \{\mathbf{x}\}$ and $i = 0$
while $i \leq I$ **do**
    Select $\mathbf{x}, \mathbf{y}$ from P randomly with replacement
    Apply recombination on $\mathbf{x}, \mathbf{y}$ to generate $\mathbf{x}', \mathbf{y}'$
    Apply bit-wise mutation on $\mathbf{x}', \mathbf{y}'$ to generate $\mathbf{x}'', \mathbf{y}''$
    for $\mathbf{z}$ in $\mathbf{x}'', \mathbf{y}''$ **do**
        if $\nexists \mathbf{u} \in P$ such that $\mathbf{u} \succ \mathbf{z}$ **then**
            $P = (P \setminus \{\mathbf{u} \in P | \mathbf{u} \prec \mathbf{z}\}) \cup \{\mathbf{z}\}$
        end if
    end for
    Check early stop
    $i = i + 1$
end while
return $\underset{\mathbf{x} \in P, \|\mathbf{x}\|_1 \leq k}{\operatorname{argmax}} \; g_1(\mathbf{x})$

S6 Computational Resources and Algorithm Runtime Evaluation

As described in the subsection ”Step 5. Optimization of Sensor Placement,” we conducted multiple independent trials in parallel to mitigate the stochastic nature of the genetic algorithm. In both the METEC experiment and the real-life case study, we ran 20 independent trials and selected the best solution among these results. The computations were performed on the Derecho high-performance computing (HPC) system from the National Center for Atmospheric Research (NCAR) [Computational and Laboratory, 2023]. For each trial, we used 4 CPUs with 16 GB of memory, similar to the configuration of a standard laptop. Figure S5 summarizes the runtimes for the METEC experiment, using detection thresholds consistent with those described in the main text, with subfigure (a) representing across-site placement and subfigure (b) representing fenceline placement. The x-axis denotes the number of sensors to install (i.e., the solution size), and the y-axis shows the runtime in minutes. Each box plot shows the runtime distribution across 20 independent trials for the corresponding sensor count. Recall that the total number of valid sensor locations on the METEC site is 146,110 for across-site placement and 21,774 for fenceline placement. The runtime generally increases with the solution size, but almost all trials remain within 1 hour for across-site placement and 15 minutes for fenceline placement, demonstrating the computational efficiency of our algorithm.

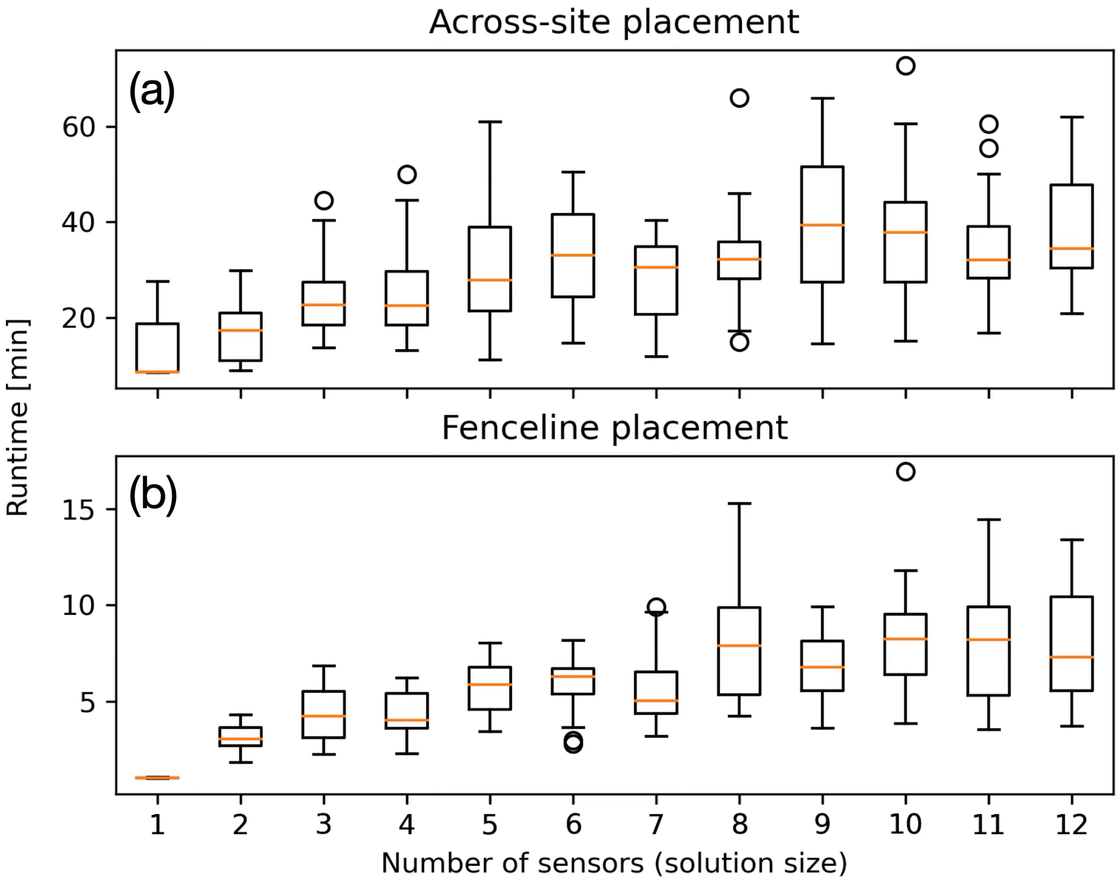

Figure S5: Runtime analysis of the PORSS optimization algorithm for the METEC experiment. (a) Box plot of runtimes for across-site sensor placement and (b) box plot of runtimes for fenceline placement, each with varying numbers of sensors from 1 to 12. Note that the vertical axes in (a) and (b) are adjusted differently for visual clarity.

S7 Performance Evaluation on Test Data

To evaluate the performance of our algorithm, we generated test data with known ground truth. Specifically, we produced random binary matrices of size 100,000 by 100,000, significantly larger than the detection matrix encountered in the study cases in this work. We assigned values of 1 to the matrices with a probability of 0.1, thereby increasing the difficulty of finding optimal solutions. Subsequently, we randomly selected 10 rows and altered them to construct the optimal solution with 100% detection coverage. We generated 30 such datasets, and for each, we conducted 10 parallel PORSS runs. Given that the best 10 rows should cover all columns, this allowed us to benchmark the solutions identified by the PORSS algorithm. As shown in Figure S6, the algorithm successfully found the best solution for all test datasets. The algorithm's success in these more challenging scenarios bolsters our confidence in its applicability to the data from METEC and a real oil and gas site discussed in the results and case study sections, respectively, and more broadly, a wide range of practical applications requiring sensor placement optimization.

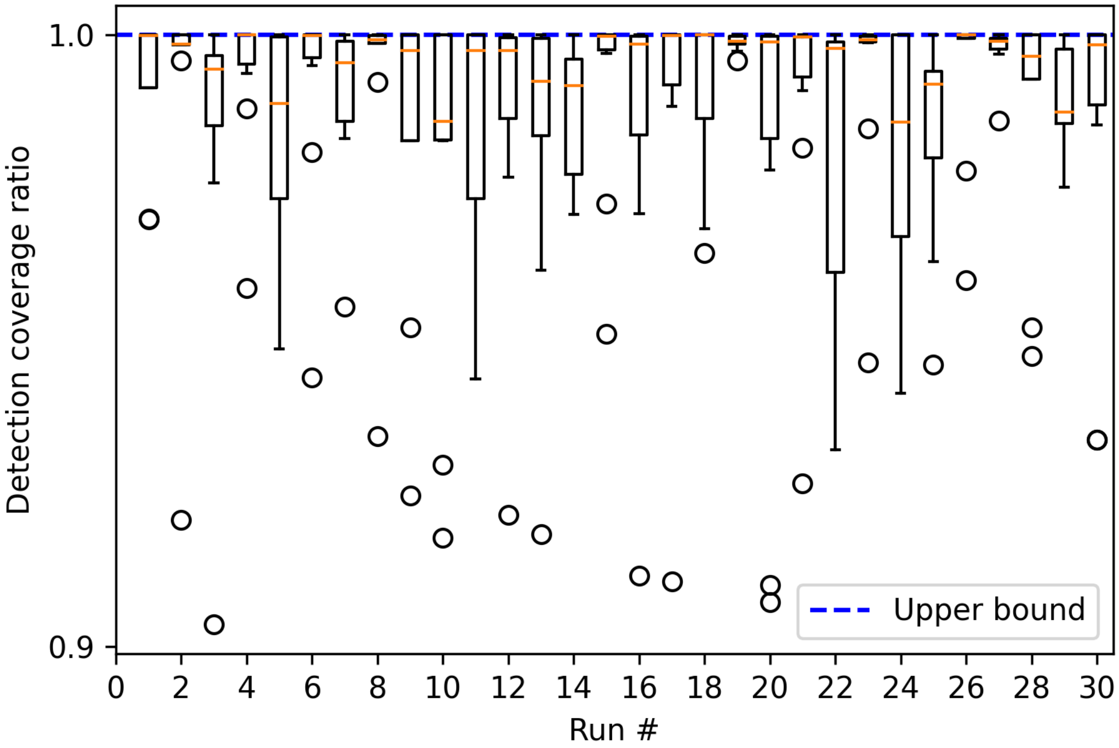

Figure S6: Illustration of the PORSS optimization performance on test data. The box plots show the detection coverage ratios on each independent test data. The blue dashed line is the ground truth of the optimal detection coverage, which is 1.0 for all cases. Note that the vertical axis ranges from 0.9 to 1.0 and does not start at 0.

S8 Optimal Sensor Placements under Different Number of Sensors

In the main text, we demonstrate examples of the optimal placement for four and eight sensors as derived by our algorithm. To provide a comprehensive overview, we have included the best sensor placements for number of sensors ranging from 1 to 12 in this section.

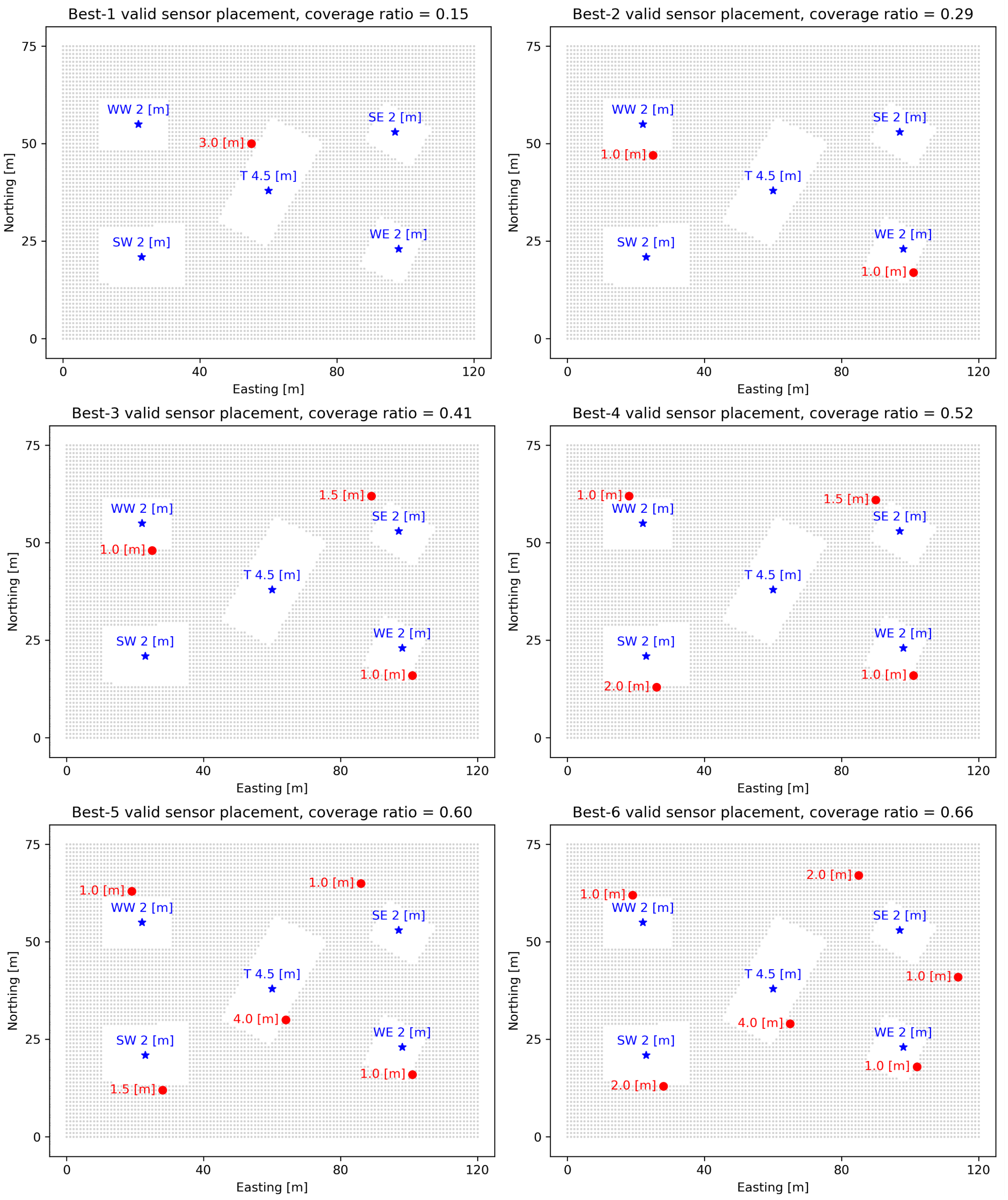

Figure S7: The optimal sensor placements derived from our algorithm for number of sensors ranging from 1 to 12. The notations are the same as in Figure 2 in the main text. (Continued on the next page.)

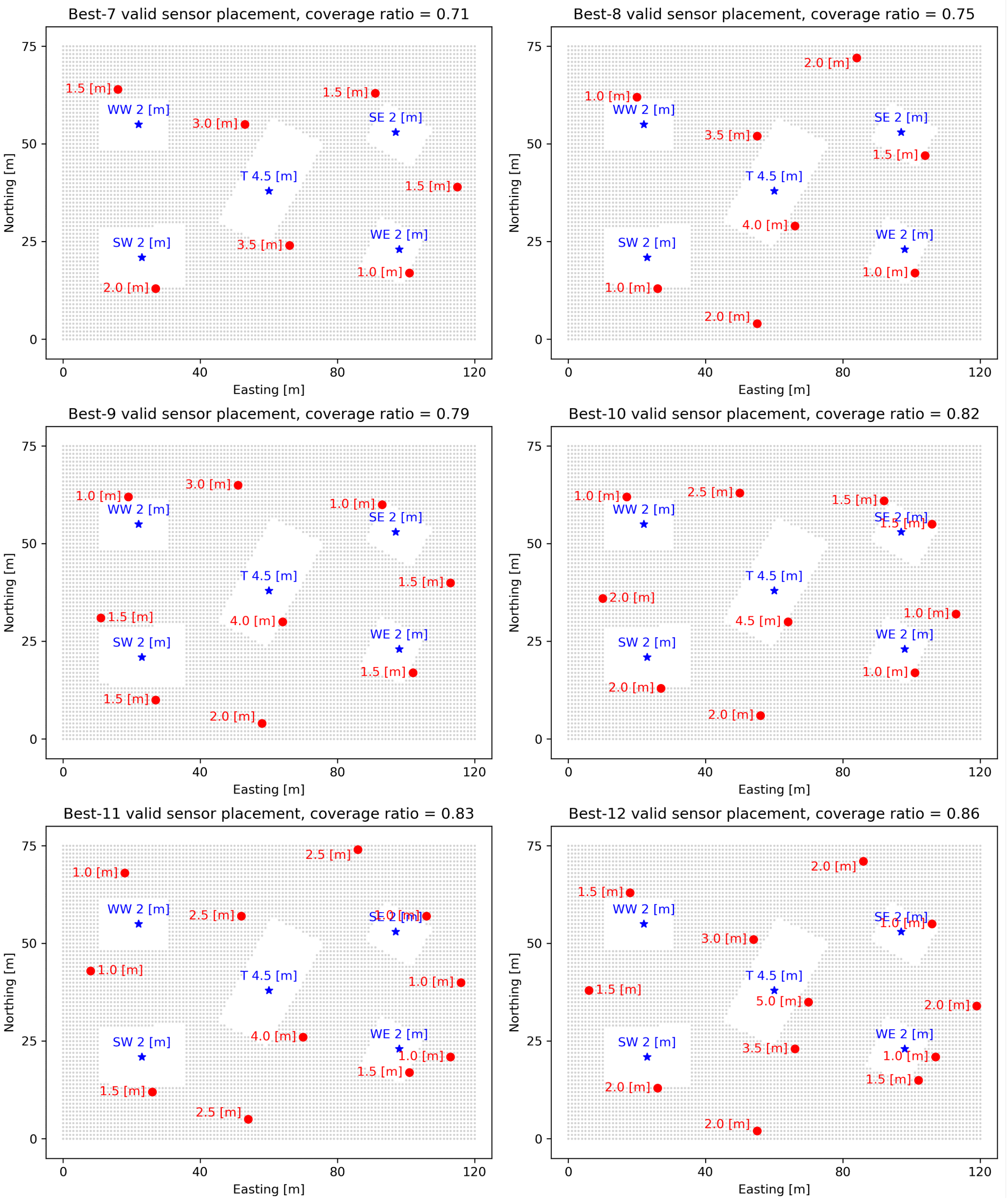

Figure S7: (Continuation of the previous page) The optimal sensor placements derived from our algorithm for number of sensors ranging from 1 to 12. The notations are the same as in Figure 2 in the main text.

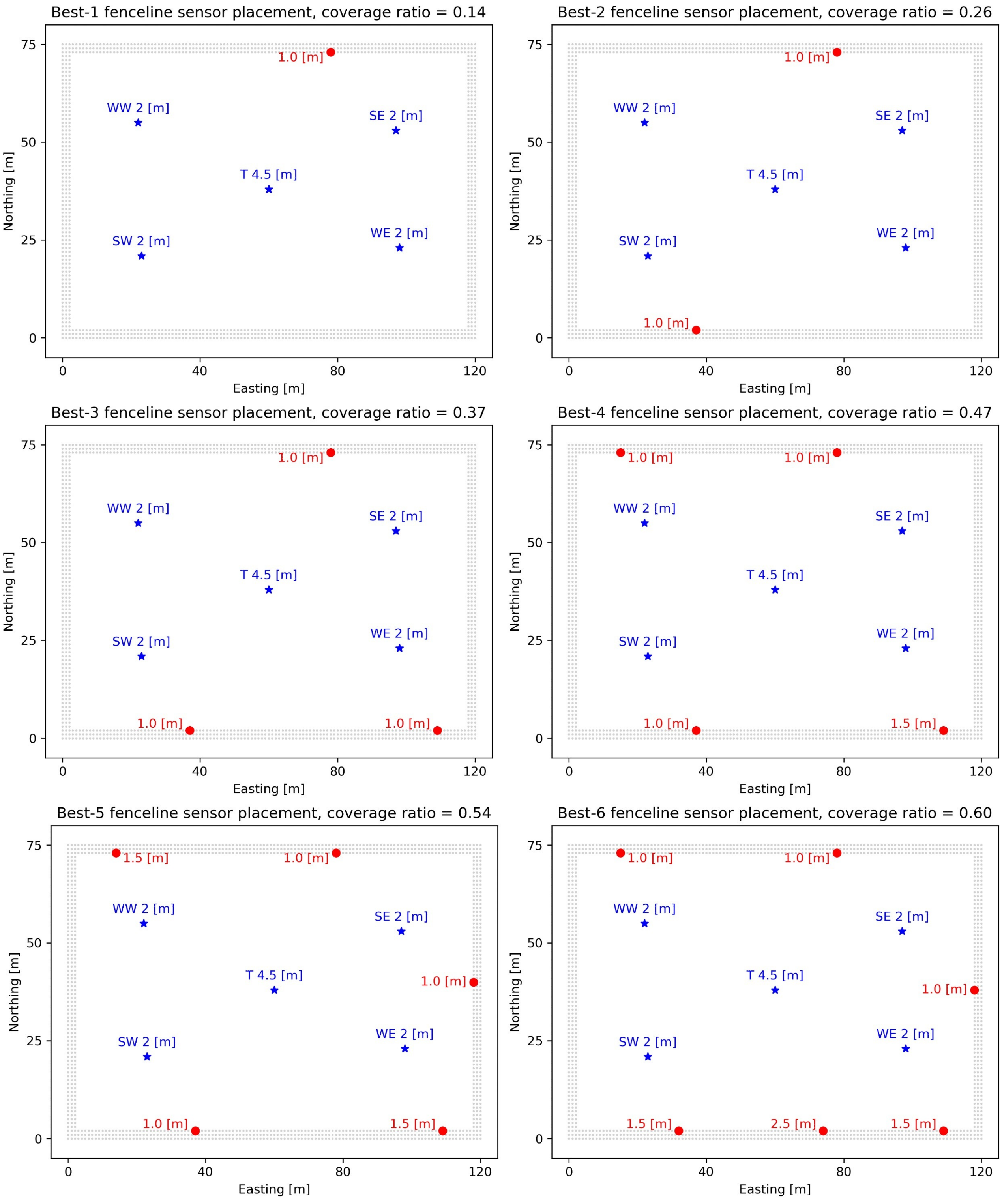

Figure S8: The optimal fence line sensor placements derived from our algorithm for number of sensors ranging from 1 to 12. The notations are the same as in Figure 2 in the main text.

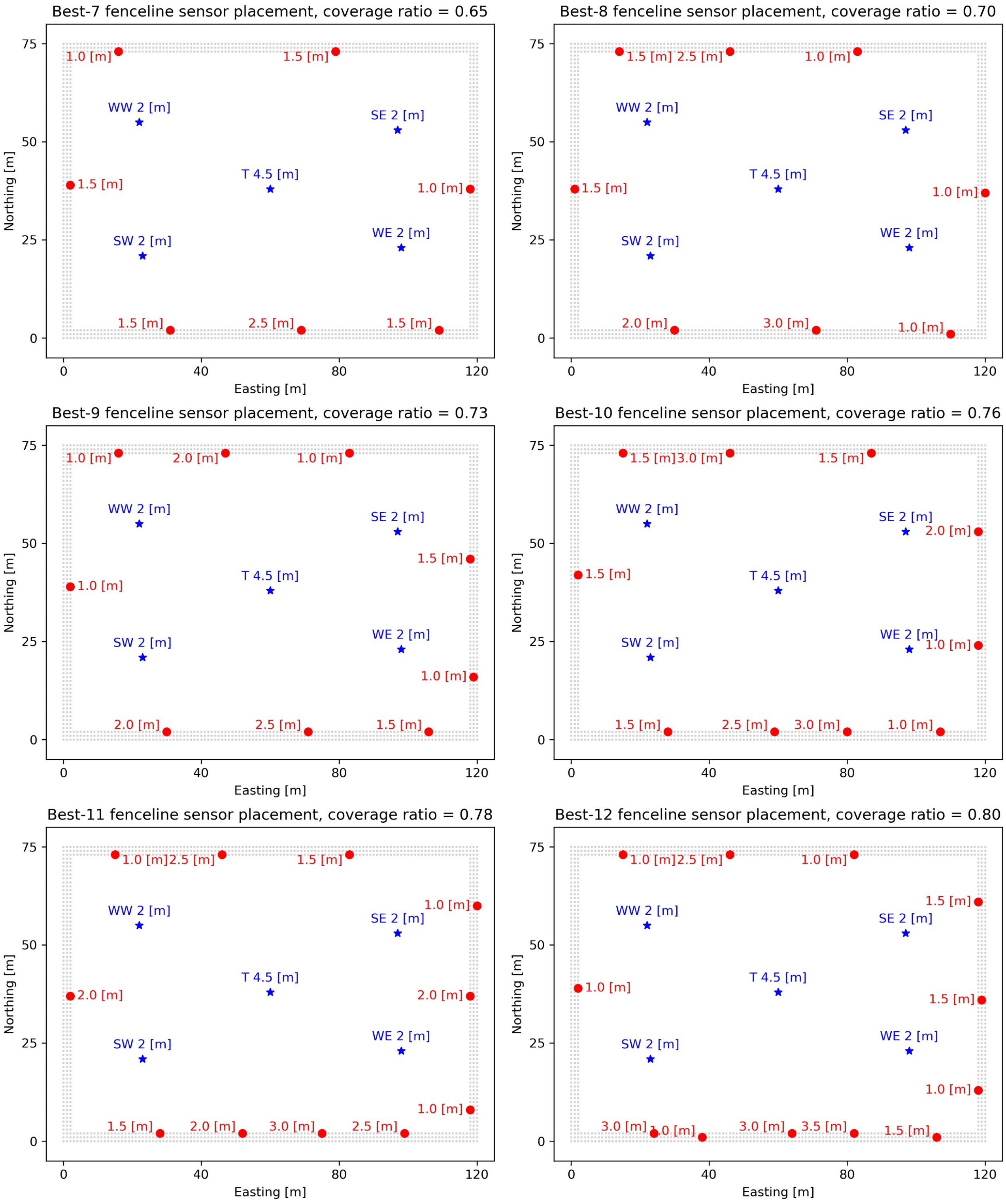

Figure S8: (Continuation of the previous page) The optimal fence line sensor placements derived from our algorithm for number of sensors ranging from 1 to 12. The notations are the same as in Figure 2 in the main text.

S9 Comparison of Detection Coverage between Continuous Monitoring Systems and Other Monitoring Technologies

In this section, we compare the detection coverage across different methane monitoring technologies, including continuous monitoring systems (CMS), optical gas imaging (OGI) cameras, aerial surveys, and satellite monitoring. In this study, detection coverage is defined as the proportion of successful event detections out of all potential emission events over a one-year period. As reported in the Results section of the main manuscript, our optimization algorithm achieves a detection coverage of 0.52 for a 4-sensor CMS placement. To provide a basis for comparison with other technologies, we first establish measurement frequencies based on typical operational practices, following the methodology of a related study on emission reduction efficiency across various monitoring technologies [Cardoso-Saldaña, 2023]. For OGI measurements, we assume a monthly survey; for aerial monitoring, we assume quarterly surveys; and for satellite monitoring, we assume daily revisits. Additionally, we assume an average of four emission events per day at a typical oil and gas production site and that all technologies can detect active emissions during their operational times, regardless of individual detection thresholds. Scaling these frequencies to a full year, we estimate detection coverage by dividing the annual measurement count by the total emission events within a year. Under this framework, the estimated detection coverage is approximately 0.009 for OGI $(12/(365 \times 4))$, 0.003 for aerial $(4/(365 \times 4))$, and 0.25 for satellite $(365/(365 \times 4))$. These rough estimates represent the upper bounds of detection coverage for each technology, assuming ideal conditions. The comparison reveals that the 4-sensor CMS achieves significantly higher detection coverage than the other methods. This advantage is further enhanced when considering methane reduction, as CMS not only enables higher detection coverage but also facilitates rapid detection and remediation of emissions.

S10 Additional Analysis from the Case Study for a Prototypical Midstream Oil and Gas Site

In the main text, the optimal overall fenceline placement for a prototypical midstream oil and gas site was determined based on emission source estimates from site operators. This section also explores the variability in the optimal configuration and coverage for different single-source emission locations. Additionally, we include ten of the best solutions found through random selection to assess the stability of our solution.

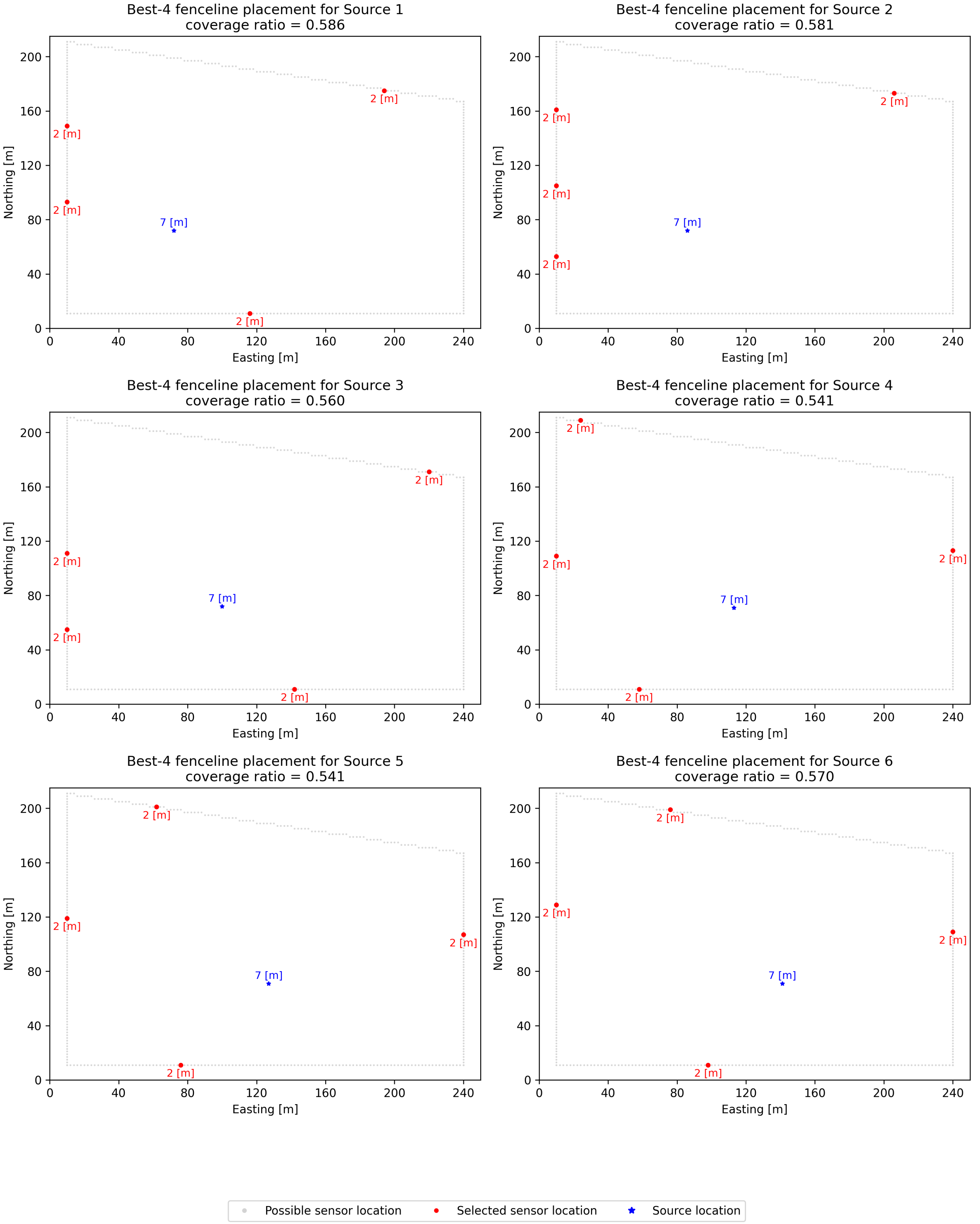

Figure S9: The optimal 4 sensor configuration for each individual source location on the prototypical midstream site. Optimal placement varies somewhat when sources are located further from the center of the site. As expected, coverage ratio is also greater for each individual source than when all sources are combined. (Continued on the next page.)

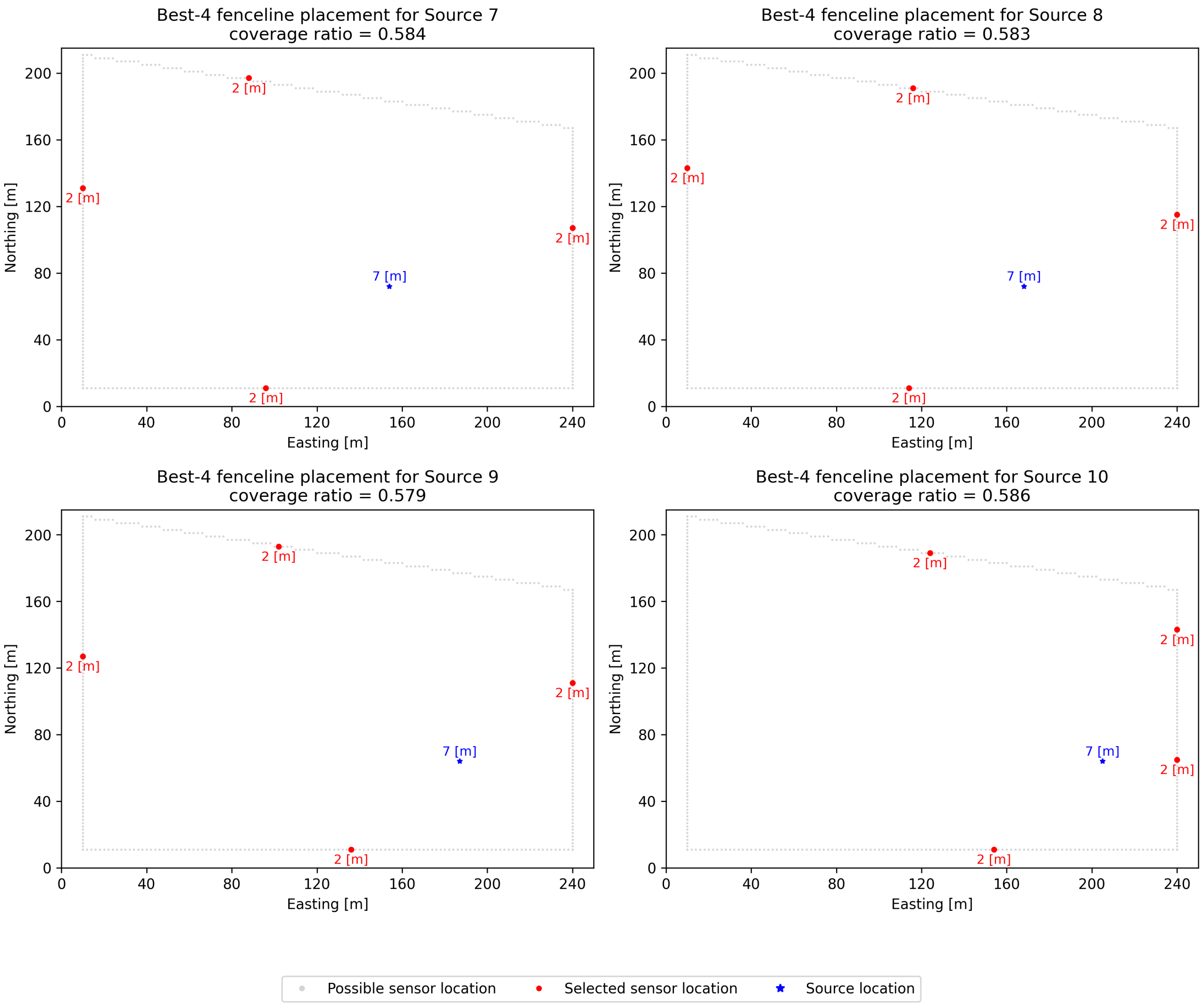

Figure S9: (Continuation of the previous page) The optimal 4 sensor configuration for each individual source location on the prototypical midstream site. Optimal placement varies somewhat when sources are located further from the center of the site. As expected, coverage ratio is also greater for each individual source than when all sources are combined.

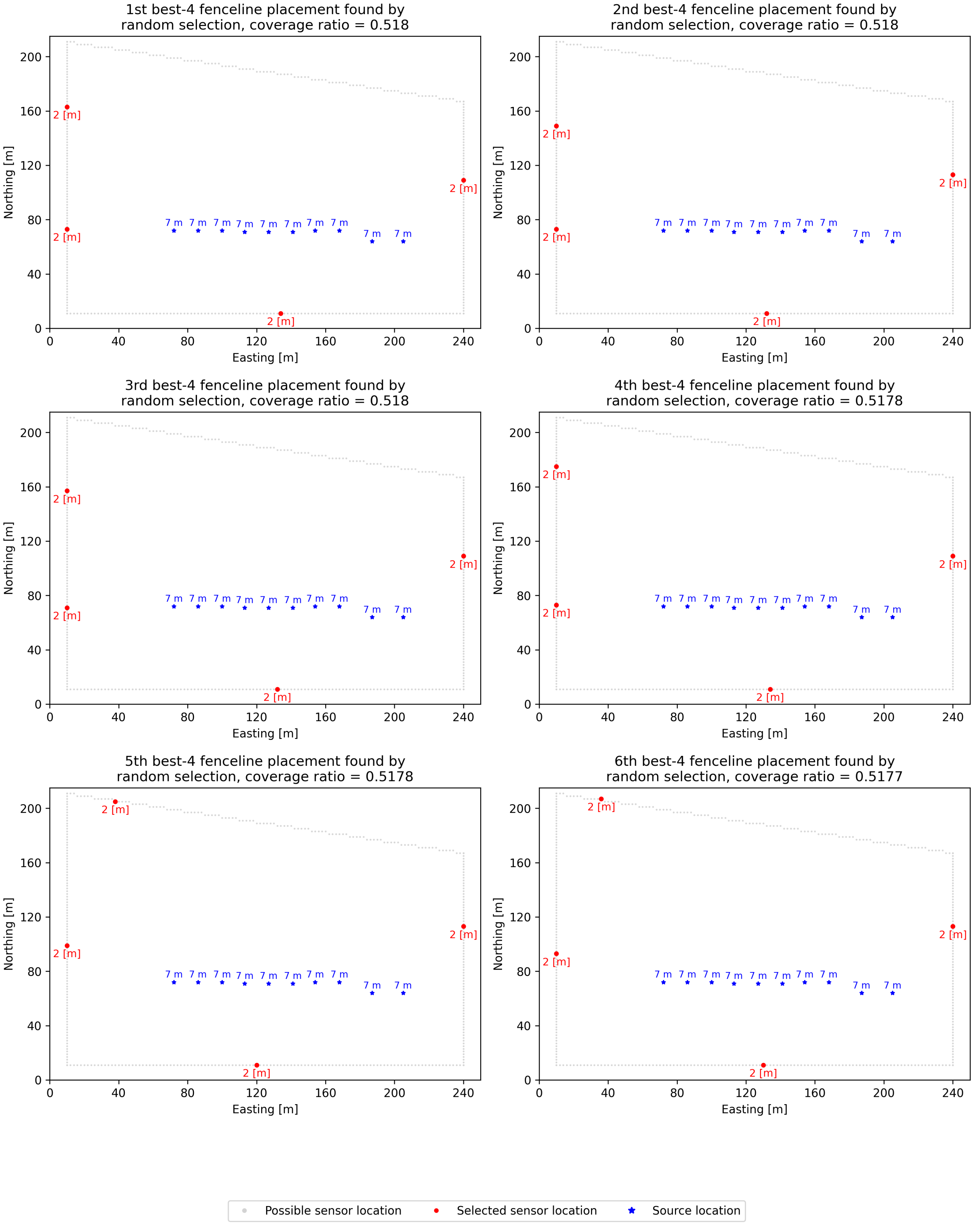

Figure S10: The top 10 best configurations found by randomly selecting 10 million 4-sensor configurations with a minimum distance of 30 m imposed between sensors. The best configuration found by random selection closely matches the configuration found by the PORSS algorithm. Configurations ranked 1, 2, 3, 4, 9 and 5, 6, 7, 8, 10 are almost identical within their respective groups, and all configurations have a very similar coverage ratio. (Continued on the next page.)

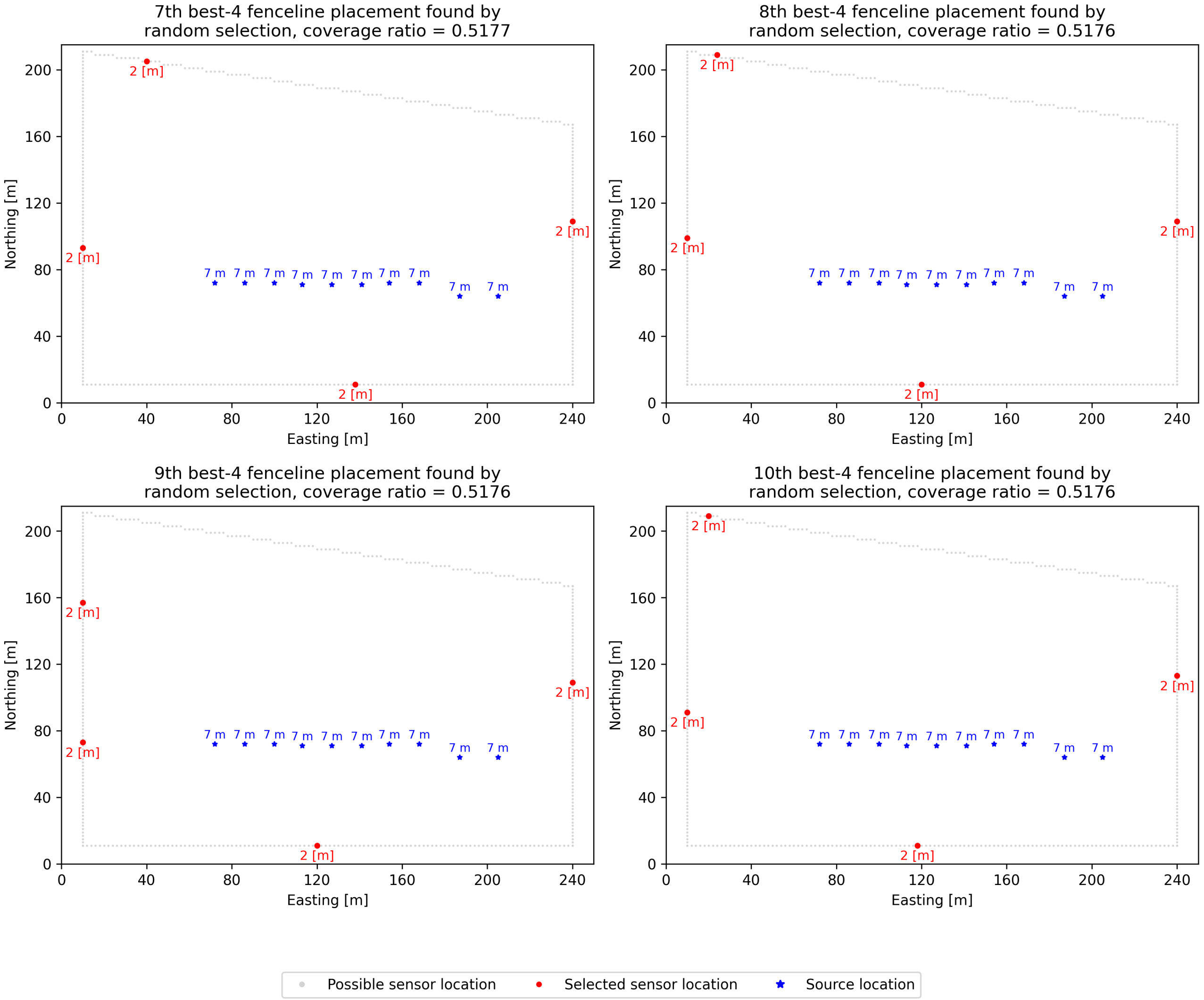

Figure S10: (Continuation of the previous page) The top 10 best configurations found by randomly selecting 10 million 4-sensor configurations with a minimum distance of 30 m imposed between sensors. The best configuration found by random selection closely matches the configuration found by the PORSS algorithm. Configurations ranked 1, 2, 3, 4, 9 and 5, 6, 7, 8, 10 are almost identical within their respective groups, and all configurations have a very similar coverage ratio.

References

[Cardoso-Saldaña, 2023] Cardoso-Saldaña, F. J. (2023). Tiered leak detection and repair programs at simulated oil and gas production facilities: Increasing emission reduction by targeting high-emitting sources. *Environmental Science & Technology*, 57(19):7382–7390.

[Computational and Laboratory, 2023] Computational and Laboratory, I. S. (2023). Derecho: HPE Cray EX System (University Community Computing).

[Jia et al., 2024] Jia, M., Fish, R., Daniels, W., Sprinkle, B., and D, H. (2024). Filling a critical need: a lightweight and fast gaussian puff model implementation. *ChemRxiv*.
